# GABAergic Medial Septal Neurons with Low-Rhythmic Firing Innervating the Dentate Gyrus and Hippocampal Area CA3

**DOI:** 10.1523/JNEUROSCI.3024-18.2019

**Published:** 2019-06-05

**Authors:** Minas Salib, Abhilasha Joshi, Linda Katona, Michael Howarth, Benjamin R. Micklem, Peter Somogyi, Tim J. Viney

**Affiliations:** Department of Pharmacology, University of Oxford, Oxford OX1 3QT, United Kingdom

**Keywords:** medial septum, dentate gyrus, hippocampus, inhibition, oscillations, rhythmicity

## Abstract

The medial septum implements cortical theta oscillations, a 5–12 Hz rhythm associated with locomotion and paradoxical sleep reflecting synchronization of neuronal assemblies such as place cell sequence coding. Highly rhythmic burst-firing parvalbumin-positive GABAergic medial septal neurons are strongly coupled to theta oscillations and target cortical GABAergic interneurons, contributing to coordination within one or several cortical regions. However, a large population of medial septal neurons of unidentified neurotransmitter phenotype and with unknown axonal target areas fire with a low degree of rhythmicity. We investigated whether low-rhythmic-firing neurons (LRNs) innervated similar or different cortical regions to high-rhythmic-firing neurons (HRNs) and assessed their temporal dynamics in awake male mice. The majority of LRNs were GABAergic and parvalbumin-immunonegative, some expressing calbindin; they innervated interneurons mostly in the dentate gyrus (DG) and CA3. Individual LRNs showed several distinct firing patterns during immobility and locomotion, forming a parallel inhibitory stream for the modulation of cortical interneurons. Despite their fluctuating firing rates, the preferred firing phase of LRNs during theta oscillations matched the highest firing probability phase of principal cells in the DG and CA3. In addition, as a population, LRNs were markedly suppressed during hippocampal sharp-wave ripples, had a low burst incidence, and several of them did not fire on all theta cycles. Therefore, CA3 receives GABAergic input from both HRNs and LRNs, but the DG receives mainly LRN input. We propose that distinct GABAergic LRNs contribute to changing the excitability of the DG and CA3 during memory discrimination via transient disinhibition of principal cells.

**SIGNIFICANCE STATEMENT** For the encoding and recall of episodic memories, nerve cells in the cerebral cortex are activated in precisely timed sequences. Rhythmicity facilitates the coordination of neuronal activity and these rhythms are detected as oscillations of different frequencies such as 5–12 Hz theta oscillations. Degradation of these rhythms, such as through neurodegeneration, causes memory deficits. The medial septum, a part of the basal forebrain that innervates the hippocampal formation, contains high- and low-rhythmic-firing neurons (HRNs and LRNs, respectively), which may contribute differentially to cortical neuronal coordination. We discovered that GABAergic LRNs preferentially innervate the dentate gyrus and the CA3 area of the hippocampus, regions important for episodic memory. These neurons act in parallel with the HRNs mostly via transient inhibition of inhibitory neurons.

## Introduction

The medial septum and diagonal band nuclei (MSDB), parts of the basal forebrain, are key relays for subcortically generated theta oscillations (Brucke et al., 1959; Vertes and Kocsis, 1997; Wang, 2002; Varga et al., 2008; Hangya et al., 2009). Disruption of the rodent MSDB results in a marked reduction in cortical theta power, affecting spatial firing fields of entorhinal grid cells and hippocampal place cell sequences and causing deficits in spatial memory (Jeffery et al., 1995; Leutgeb and Mizumori, 1999; McNaughton et al., 2006; Brandon et al., 2011; Wang et al., 2015; Zutshi et al., 2018). Rhythmically burst-firing “theta cells” recorded in the MSDB have been hypothesized to provide rhythmic entrainment of hippocampal target neurons (Petsche et al., 1962; Ford et al., 1989; Sweeney et al., 1992; Borhegyi et al., 2004; Simon et al., 2006). How this is implemented is unclear because the MSDB contains GABAergic, cholinergic, and glutamatergic neurons (Kimura et al., 1980; Freund, 1989; Hajszan et al., 2004; Colom et al., 2005) with various developmental origins (Wei et al., 2012). The GABAergic component innervates exclusively GABAergic neurons in the hippocampus (Freund and Antal, 1988). Aside from innervating the entire temporal cortex, MSDB neurons also innervate the prefrontal cortex, parietal cortex, supramammillary nucleus, ventral tegmental area, nucleus incertus, pontine central gray, dorsal and median raphe nuclei, and several other subcortical areas (Swanson and Cowan, 1979; Gritti et al., 2003; Gonzalez-Sulser et al., 2014; Unal et al., 2015; Fuchs et al., 2016; Desikan et al., 2018; Agostinelli et al., 2019). Given this diversity, what is the relationship among firing patterns, target brain regions, and synaptic target neurons?

High-rhythmic-firing medial septal neurons (HRNs) include Teevra cells, the most rhythmic subpopulation of mouse MSDB neurons, providing selective GABAergic input to axo-axonic cells and cholecystokinin-immunopositive GABAergic neurons in hippocampal CA3 (Joshi et al., 2017). In contrast, another kind of HRN, the rhythmically bursting GABAergic orchid cells, avoid the hippocampus and project mainly to the dorsal presubiculum and caudodorsal entorhinal cortex (Viney et al., 2018). Because rhythmicity facilitates neuronal coordination in cognition, these two kinds of parvalbumin-immunopositive (PV^+^) HRNs are likely to be major contributors to the coordination of neuronal activity within and across these regions. In rodents, CA3 principal cells preferentially fire on the descending phase of theta recorded in dorsal CA1 (CA1d) stratum pyramidale (SP) (Mizuseki et al., 2009). Because Teevra cells preferentially fire at the CA1d theta trough and axo-axonic cells at the peak (Viney et al., 2013; Joshi et al., 2017), CA3 principal cells are effectively disinhibited on the descending/trough phase of each cycle. Furthermore, axo-axonic cells reduce their firing during sharp-wave-associated ripple oscillations (SWRs) (Viney et al., 2013), disinhibiting pyramidal cell axon initial segments, which was originally proposed as a mechanism for SWR initiation (Buzsáki, 1986).

Dentate gyrus (DG) principal cells, the granule and mossy cells, also fire preferentially on the descending phase of CA1d theta (Mizuseki et al., 2009) and are modulated during SWRs (Penttonen et al., 1997). Intriguingly, both Teevra and orchid HRNs avoid the DG, an area that also receives dense innervation from the MSDB (Freund and Antal, 1988), but the firing dynamics of these particular afferents are unknown (Bao et al., 2017). In addition to CA3 pyramidal neurons (Li et al., 1994), local GABAergic interneurons (Han et al., 1993; Szabo et al., 2017) and SWR-suppressed MSDB neurons (Unal et al., 2018) provide shared input to the DG and CA3. The MSDB also receives GABAergic input from the DG and hippocampus (Tóth et al., 1993; Jinno et al., 2007; Mattis et al., 2014; Yuan et al., 2017), which may contribute to septohippocampal coordination (Wang, 2002). We asked whether low-rhythmic-firing neurons (LRNs) of the MSDB (Ford et al., 1989; King et al., 1998) could also be GABAergic and investigated how they contribute to septocortical circuits in parallel with HRNs by extracellularly recording them in awake mice followed by juxtacellular labeling to reveal their target regions and target neurons.

## Materials and Methods

### 

#### Surgical procedures

All procedures involving experimental animals were approved by the Department of Pharmacology Animal Welfare and Ethical Review Body under approved personal and project licenses in accordance with the Animals (Scientific Procedures) Act, 1986 (UK) and associated regulations. The following adult mice were used: 160 male C57BL7/J mice for head-plate implantation (Charles River Laboratories; 24–37.5 g; this included all 120 mice from Viney et al., 2018, and 40 new mice); 3 VGAT^Cre^ mice for viral tracing (The Jackson Laboratory, Bar Harbor; stock #016962; kind donation from Prof. William Wisden; 21–37.5 g; 1 male, 2 females); 1 male PV^Cre^ mouse for viral tracing (The Jackson Laboratory; stock #008069; ∼28 g). Mice were maintained on a 12/12 h light-dark cycle (lights on during the day) and before surgery housed in groups of up to four within individually ventilated cages. Mice were anesthetized with isoflurane (IsoFlo; Abbott) followed by a subcutaneous injection of opioid analgesic buprenorphine (Vetergesic, 0.1 mg/kg), and maintained with 1–3% (v/v) isoflurane. The scalp was clipped and mice were fixed to a stereotaxic frame (Kopf Instruments) using ear bars and a jaw bar. Ocular lubricant was applied and small volumes of the nonsteroidal anti-inflammatory analgesic meloxicam (Metacam; Boehringer Ingelheim) were injected into the scalp. Under aseptic conditions, an incision was made along the scalp at the midline and the skull was exposed.

##### Head-plate implantation.

Two M1 screws (Precision Technology Supplies) were fixed into the skull above the cerebellum, one of which was used as the electrical reference. Another screw was fixed at 1.50 mm anterior and 1.70 mm lateral of bregma and used as a frontal cortical EEG. A second EEG screw was placed over the retrosplenial cortex/V1 at −2.10 mm posterior and −2.50 mm lateral of bregma (for *n* = 96 mice). A machined glass-reinforced plastic head plate (either a 0.7 g or 1.1 g version, custom made at the Department of Physics, Oxford University) was positioned over the screws and bone cement (Zimmer Biomet) was used to fix the head-plate and screws to the skull. Craniotomies were made above the MSDB (0.85 mm anterior and 0 mm lateral of bregma) and right CA1d (2.50 mm posterior and 1.70 mm lateral of bregma). Craniotomy sites were covered using silicone (Smooth-On) and mice were left to recover (typically 1–2 d). For some experiments (*n* = 31 mice), craniotomies were instead performed during a second surgery using the same anesthesia regime as above.

##### Viral tracing.

After performing a small craniotomy at 0.86 mm anterior and 0.39 mm lateral of bregma, a glass pipette (tip diameter: 12–20 μm, 5 μl; Harvard Apparatus) was lowered at a 5° lateromedial angle to 3.75 mm ventral of the dura mater into the MSDB. Anterograde Cre-dependent AAV2-CAG-FLEX-ArchT-GFP (*n* = 4 mice; 400 nl/mouse; UNC Vector Core) or pAAV2-EF1a-DIO-EYFP (*n* = 3 mice; same mice used in Unal et al., 2015) was pressure injected using a 1 μl syringe at a rate of ∼100 nl/min. Mice were perfuse-fixed >28 d after injections to ensure optimal viral expression.

#### *In vivo* neurophysiology

##### Acute silicon probe recordings.

Data were obtained from four mice used in Joshi et al. (2017). Briefly, head-restrained mice were trained to run on an air-flow suspended Styrofoam ball (jetball). Medial septal units were recorded using a two-shank acute silicon probe (150 μm intershank distance; two tetrodes per shank; 25 μm spacing between contacts within a tetrode; NeuroNexus) connected to an RA16-AC preamplifier (Tucker-Davis Technologies). Recordings were then digitally band-pass filtered (0.8–5 kHz) and neuronal spikes were detected using a threshold-crossing-based algorithm. Detected spikes were automatically sorted using the algorithm implemented in KlustaKwik (Kadir et al., 2014), followed by manual adjustment of the clusters (Csicsvari et al., 1999) to obtain well isolated single units based on cross-correlations, spike waveform, and refractory periods.

##### Extracellular recordings and juxtacellular labeling.

Experiments were performed as described previously (Viney et al., 2018). Briefly, experiments were conducted in a dedicated recording room during the light phase, typically 1–3 d after the craniotomies. Mice were habituated to a circular treadmill, a running disc (Fast Trac; LBS), or a Frisbee (radius 15 cm) below a stereotaxic frame and attached to a head-restraint device (custom made at the Department of Physics, Oxford University) secured to a heavy-duty frame (model 1430; David Kopf Instruments). Two separate glass electrodes filled with 3% neurobiotin (w/v) in 0.5 m NaCl (10–24 MΩ) were advanced into the brain, targeting SP of CA1d at a 10° posteroanterior angle (sometimes filled only with 0.5 m NaCl) and the midline dorsal MS (0° angle, near or directly through the sagittal sinus). For animals MS83, MS86, MS103, MS104, and MS109, 10% biotinylated dextran amine (BDA, 3000 MW; Life Technologies) was used instead of neurobiotin (*n* = 5/14 reported labeled cells; see Table 2). Signals were amplified ×1000 (ELC-01MX, BF-48DGX and DPA-2FS modules; npi Electronic). Both wide-band (0.3 Hz to 10 kHz) and band-pass-filtered (action potentials, 0.8–5 kHz; LFPs, 0.3–500 Hz) signals were acquired in parallel and digitized at 20 kHz (Power1401; Cambridge Electronic Design). HumBugs (Digitimer) were used to remove 50 Hz noise. A video camera was used to monitor behavior and, for experiments using the circular treadmill, speed was recorded using an Arduino, as described previously (Viney et al., 2018). In other experiments, an accelerometer was placed on the wheel to detect wheel movement and in one experiment (animal TV68) an electromyogram was used to help detect movement of the animal (from the neck muscle). Data were recorded using Spike2 software (Cambridge Electronic Design). Extracellularly recorded neurons in the MSDB were juxtacellularly labeled with 200 ms positive current pulses (Pinault, 1996), followed by a recovery period of 4–10.5 h for neurobiotin and 20–48 h for BDA. Multiple neurons were recorded in each animal. Because neurobiotin degrades within 24 h, we sometimes attempted to label neurons with neurobiotin on different days for the same animal. We have only included recordings that we could match to a single labeled neuron. Due to insufficient evidence to match the recordings with the recovered neurons in animals MS09 and MS53, we excluded the physiology in both brains from our analysis.

##### Targeting single MSDB low-rhythmic neurons.

Following the recovery of neurons TV68a, TV77q, and TV78l and the observation that they were distinct from orchid cells (Viney et al., 2018), we probed the MSDB for neurons exhibiting the following parameters: a low burst incidence during immobility (see below), a strong reduction in firing during SWRs, and a low rhythmicity and change in firing frequency during locomotion. We confirmed the glass electrode to be in the MSDB by identifying known HRN MSDB cells 2500–4000 μm from the brain surface. We recorded a total of 1182 single MSDB neurons and recovered 67 that were juxtacellularly labeled.

#### Histology

##### Tissue processing.

Mice were deeply anesthetized with sodium pentobarbital (50 mg/kg, i.p.) and transcardially perfused with saline followed by 4% paraformaldehyde (PFA), 15% v/v saturated picric acid, and 0.05% glutaraldehyde in 0.1 m phosphate buffer (PB), pH 7.4 (except 2% PFA for 1 PV^Cre^ mouse). After washing in 0.1 m PB, 70–100 μm coronal sections were cut using a Leica Microsystems VT 1000S vibratome and stored in 0.1 m PB with 0.05% sodium azide at 4°C. Streptavidin-conjugated fluorophores were used to visualize neurobiotin-labeled neuronal processes within tissue sections previously permeabilized by Tris-buffered saline (TBS) with 0.3% Triton X-100 (TBS-Tx) or through rapid 2–5× freeze–thaw (FT) over liquid nitrogen (cryoprotected in 20% sucrose in 0.1 m PB). For light microscopic visualization, analysis, and 3D neuronal reconstruction, TBS-Tx- or FT-processed sections were processed using horseradish peroxidase-based diaminobenzidine (DAB) reactions as described previously (Viney et al., 2013). For neurons labeled with BDA, we observed a relatively poor tissue processing result when using TBS-Tx compared with TBS.

##### Immunohistochemistry.

For the molecular identification of labeled neurons and their postsynaptic target neurons, immunohistochemistry was performed as described previously (Viney et al., 2013, 2018; Unal et al., 2015). Specificity information for primary antibodies is provided in Table 1. To test the immunoreactivity of multiple markers on the same neurons (e.g., on postsynaptic targets of septocortical neurons), we used an iterative strategy based on area-dependent marker frequency, subcellular localization, colocalization probability, antibody species, and fluorophores, as described previously (Viney et al., 2018).

**Table 1.** Information for primary antibodies

Molecule	Host	Dilution	Source	Specificity information	RRID
Calbindin (CB)	Rb	1:5000	Swant, catalog #CB-38 (lot #5.5)	No signal in knockout. Mouse knockout: Airaksinen et al., 1997; characterization in rat hippocampus: Sloviter, 1989.	AB_2721225
Calretinin (CR)	Rb	1:500–1:1000	Swant, catalog #7699/^3^H (lot #18299)	Western blot supplied by Swant. No signal in knockout animals: Schiffmann et al., 1999.	AB_10000321
Calretinin (CR)	Gt	1:1000	Swant, catalog #CG1	Antibody does not label the brain of CR knock-out mice (manufacturer's technical information).	AB_10000342
Choline acetyltransferase (ChAT)	Gt	1:500	Chemicon, catalog #AB144P	Western blot (manufacturer's technical information).	AB_2079751
Gephyrin	Ms	1:500	Synaptic Systems catalog #147 021	Western blot; band at 93 kDa. No Geph7a signal in knockout. Co-purifies with glycine receptor. Mouse knockout: Feng et al., 1998. Antibody generation: Pfeiffer et al., 1984.	AB_2232546
GFP	Ck	1:500	Aves Labs, catalog #GFP-1020	Antibody does not label the brain of wild-type control mice.	AB_10000240
Muscarinic acetylcholine receptor M2 (M2R)	Rt	1:400	Chemicon, catalog #MAB367 lot #2383976	Distinct from other subtypes; characterized by Levey et al., 1991.	AB_94952
Metabotropic glutamate receptor 1a (mGluR1a)	Gp	1:500–1:1000	Frontier Institute, catalog #mGluR1a-GP-Af660	Characterized by Tanaka et al., 2000.	AB_2531897
Neuronal Ca^2+^-binding protein 1 (NECAB 1)	Ms	1:500	Abnova, catalog #H00064168-B01P	Western blots; as expected, no cross-reactivity with NECABs 2 and 3. Mouse brain and antibody generation: Sugita et al., 2002.	AB_2149170
Neuronal nitrogen oxide synthase (nNOS)	Rb	1:1000	EMD Millipore, catalog #AB5380	Does not cross react with iNOS or eNOS. No signal in knockout for rabbit antibody (Zymed/Invitrogen) of same mass. Mouse knockout and similar rabbit antibody: Gyurko et al., 2002.	AB_91824
Substance P receptor (NK1R)	Rb	1:500	Millipore, catalog #AB5060, lot #LV1525037	Characterized by Shigemoto et al., 1993.	AB_2200636
Parvalbumin (PV)	Rb	1:1000	Swant, PV-28	No signal in knockout, shown by Swant. Similar to other parvalbumin antibodies. Mouse knockout: Schwaller et al., 1999.	AB_2315235
Parvalbumin (PV)	Gt	1:1000	Swant, catalog #PVG-214, lot #3.6	No signal in knockout, shown by Swant. Similar to other parvalbumin antibodies. Mouse knockout: Schwaller et al., 1999.	AB_2313848
Parvalbumin (PV)	Gp	1:5000	Synaptic Systems, catalog #195 004, lot #5	Western blot; abolished by pre-absorption with recombinant parvalbumin. Same labeling as Synaptic Systems rabbit antibody 195 002 and Swant monoclonal antibody 235. Rat hippocampus: Kosaka et al., 1987, Sloviter, 1989. Mouse knockout: Schwaller et al., 1999.	AB_2156476
Purkinje cell protein 4 (PCP4)	Rb	1:1000	Santa Cruz, catalog #sc-74816, lot #G0814	Characterized by San Antonio et al., 2014.	AB_2236566
Pro-cholecystokinin (pro-CCK)	Rb	1:500	April 2005 gift (similar to Frontier Institute, CCK-pro-Rb-Af350)	Similar labeling to two noncommercial antibodies characterized by Sloviter and Nilaver, 1987, Morino et al., 1994.	AB_2571674
Regulator of G-protein signaling 14 (RGS-14)	Ms	1:500–1:1000	NeuroMab, catalog #75–170, clone N133/21	Characterized by manufacturer (see datasheet).	AB_2728538
SATB1 (N-14)	Rb	1:200	Abcam, catalog #ab70004	No signal in knockout for a similar rabbit antibody. Western blot, different band to SATB2 at mouse P1. Neuron specificity shown by NeuN colocalization. Original rabbit antibody: Dickinson et al., 1992. Mouse cortex: Huang et al., 2011. Mouse knockout: Balamotis et al., 2012.	AB_1270545
SATB1 (N-14)	Gt	1:200–1:250	Santa Cruz, catalog #sc-5989	Similar labeling to rabbit antibody ab70004. Mouse cortex: Huang et al., 2011. Mouse knockout: Balamotis et al., 2012.	AB_2184337
Secretagogin	Gt	1:1000	R&D Systems, catalog #AF4878 lot #CASX0115091	Characterized by Mulder et al., 2009.	AB_2269934
SMI32 (neurofilament H non-phosphorylated)	Ms	1:1000	Covance, catalog #SMI-32R lot #14835102	Similar to immunoreactivity characterized in primates by Campbell and Morrison, 1989, and in rats by Ouda et al., 2012.	AB_509997
Somatostatin (SOM)	Ms	1:500	GeneTex, catalog #gtx71935 clone SOM-018	Same labeling as a rat antibody Chemicon MAB354. No signal in preabsorption test for rat antibody. Mouse hippocampus, rat antibody: Jinno and Kosaka, 2000. Rat antibody test: Kubota et al., 2011.	AB_383280
Somatostatin (SOM)	Rb	1:50	GenWay Biotech, catalog #18-783-76392	Similar distribution and subcellular localization as Ms anti-SOM.	AB_1027453
Vesicular acetylcholine transporter (VAChT)	Gt	1:1000	Millipore catalog #ABN100	This antibody recognizes the C-terminus of vesicular acetylcholine transporter (VAChT).	AB_2630394
Vesicular GABA transporter (VGAT)	Gp	1:500	Synaptic Systems, catalog #131 004	Similar to C- and N-terminal fusion protein antibodies. Former gave additional unknown lower mass band in brain extract but same immunoreactivity as specific latter antibody; see rat cortex: Chaudhry et al., 1998. Similar rabbit antibody generation: Takamori et al., 2000.	AB_887873
Vesicular glutamate transporter 2 (VGLUT2)	Gp	1:2000	Synaptic Systems, catalog #135 404, lot #135404/16	Similar to immunoreactivity characterized in mouse hippocampus by Herzog et al., 2006.	AB_887884

Rb, Rabbit; Gt, goat; Ms, mouse; Ck, chicken; Rt, rat; Gp, guinea pig.

##### Microscopy.

Confocal microscopy (LSM 710; Carl Zeiss, ZEN 2008 version 5.0) was used to document identified neurons and their targets, as described previously (Viney et al., 2013; Unal et al., 2015). Overviews of multichannel multiround sections tested with immunohistochemistry were acquired using widefield epifluorescence either on the AxioImager.Z1 (Carl Zeiss, AxioVision 2009 version Rel.4.8.1) or on a Leitz DMRB microscope (Leica, Openlab version 5.5.0).

##### Identification of target neurons using DAB.

When immunofluorescence signal quality of neurobiotin-labeled axons was suboptimal, we combined this method with DAB visualization to identify MSDB axon terminals and their postsynaptic targets. Brain sections have been immunoreacted sequentially and, after each round, areas of interest with putative axon collaterals have been tile scanned at 20× [PL Fluotar 0.5 numerical aperture (NA)] magnification using either wide-field epifluorescence or confocal microscopy. The process was concluded by taking low- and high-resolution (α Plan-Apochromat 100×/1.46 NA oil objective) light micrographs of the DAB reactions. Sequential image stacks (typically 40–50 optical sections) of the same section were then matched up using blood vessels and endogenous biotin signal as references and neurons postsynaptic to the DAB visualized axon terminals were identified and their immunoreactivity determined for the tested molecular markers.

#### Electrophysiological and behavioral data analysis

Data were analyzed in Mathematica (Wolfram Research), MATLAB (The MathWorks), and Spike2. Movement periods (including changes in posture, limb movements, and locomotion) were detected by the combination of video, wheel activity, and in some cases EMG or accelerometer. Only data acquired before juxtacellular labeling were used for analysis.

##### LFPs and oscillations.

The position of the CA1d LFP recording was estimated based on the polarity of sharp waves (Buzsáki, 1986) and the presence of ripples (Buzsáki et al., 2003). Both strata oriens and pyramidale contained positive sharp waves and stratum radiatum (SR) contained negative sharp waves. The upper part of superficial SP consisted of both positive and negative sharp waves. Theta periods were initially detected by filtering the CA1d LFP for theta (5–12 Hz) and delta (2–4 Hz), computing a power ratio, and then manually adjusting them. Theta phase was calculated by linear interpolation between troughs of the band-pass-filtered theta oscillations, with 0° and 360° set as the troughs. Rayleigh test was used to test for uniformity of circular phase distributions. Mean phase and mean vector length were used as measures of the preferred phase and coupling strength, respectively. Phase histograms were smoothed by convolving with a Gaussian. 'Theta off' in Table 2 refers to the percentage of theta cycles (trough to trough) that contained zero spikes for a given single neuron recording. Gamma oscillations were detected by band-pass filtering the CA1d wide-band LFP for slow-gamma (32–39 Hz) and mid-gamma (50–80 Hz) frequencies and selecting cycles that were >1 SD above the mean cycle amplitude.

**Table 2.** Properties of identified low-rhythmic neurons

Cell name	MS13c	MS109o	MS83d	MS68a	TV78l	MS16d	MS09	TV77q	MS53	TV68a	MS10	MS86b	MS33a	MS104e
Recovery	*^a^*					*^b^*	*^c^*		*^c^*		*^a^*			
Target hemisphere	Left	Left	Right	Left	Left	Left	Right	Left	Left	Right	Left	Left	Right	Right
Proportion of DG terminals	∼99%	u	u	∼90%	98%	u	62%	30%	∼20%	21%	u	u	u	0%
Innervated areas	DG, u	u	u	DG, CA3	DG, CA3	DG, u	DG, CA3	CA3, DG	CA3, DG, CA2, CA1, SUB, MS	CA3, DG, CA2, CA1, SUB, MS	CA3, DG, u	u	CA3, CA1, SUBd	CA1, RSg, SUBd, PrSd
Immunoreactivity														
PV	a^−^	a^−^	x	a^−^	tads^−^	a^−^	a^−^	tads^−^	ad^−^	ads^+^	a^+^	a^−^	x	d^+^
Calbindin	a^+^	ads^+^	x	a^+^	d^−^	a^−^	a^−^	ad^−^	ad^−^	a^−^	a^−^	x	x	as^−^
SATB1	u	n^−^	x	n^−^	n^+^	u	u	n^+^	u	n^+^	u	n^−^	x	n^+^
mGluR1a	d^+^	x	x	d^−^	ds^+^	u	u	ds^+^	d^+^	ds^+^	u	ds^+^	x	ds^+^
VGAT	x	u	u	u	t^+^	u	t^+^	t^+^	x	t^+^	x	u	u	t^+^
VGLUT2	u	u	u	u	t^−^	u	t^−^	u	u	u	u	u	u	u
VACHT	u	u	u	u	t^−^	u	t^−^	u	u	u	u	u	u	u
NK1R	d^−^	u	u	u	d^−^	u	u	ds^−^	d^+^	s^+^	u	u	u	u
PCP4	d^−^	u	u	u	d^+^	u	u	ds^+^	u	u	u	u	u	x
Calretinin	d^−^	u	u	u	tad^−^	u	u	ad^−^	u	a^−^	u	u	u	u
ChAT	a^−^	u	u	u	u	a^−^	u	a^−^	ad^−^	u	u	u	x	u
Labeling strength	++	+	+	++	+++	++	++	++	++	++	+	+	+	+++
Firing patterns														
Rhythmicity index M	0.06	0.03	0.04	0.14	(low)	0.01	u	0.03	u	0.04	0.04	0.02	0.04	0.02
Firing rate M (Hz)	52 ± 9	50 ± 7	41 ± 8	24 ± 5	23 ± 11	31 ± 6	u	31 ± 13	u	11 ± 10	19 ± 9	18 ± 10	25 ± 8	54 ± 16
Firing rate IM (Hz)	54 ± 10	61 ± 13	38 ± 12	27 ± 9	22 ± 6	36 ± 10	u	16 ± 8	u	24 ± 12	15 ± 7	16 ± 11	14 ± 6	31 ± 19
Burst incidence M (Hz, 20)	4.6	4.9	2.6	1.0	0.3	1	u	1.5	u	0.5	1.8	0.4	0.3	4.7
Burst incidence IM (Hz, 20)	4.5	4.7	2.4	2.0	0.5	2.3	u	0.9	u	1.4	0.5	0.4	0.1	2.2
Burst incidence M (Hz, 40)	3.1	3.5	4.1	2.6	1.6	3	u	2.2	u	1	0.8	1.1	0.7	2.0
Burst incidence IM (Hz, 40)	2.7	2.2	3.5	2.76	1.8	3.2	u	1.5	u	2.3	1.1	1.1	0.5	2.2
Interburst interval M	226, 251	222, 206	206, 150	273, 254	537, 413	252, 194	u	197, 207	u	417, 1282	326, 347	778, 807	272, 190	362, 364
Interburst interval IM	297, 244	338, 379	246, 160	312, 220	394, 394	266, 196	u	446, 616	u	309, 275	588, 949	563, 963	854, 1613	301, 306
Intraburst frequency M	74 ± 17	68 ± 14	74 ± 19	88 ± 35	68 ± 27	65 ± 18	u	81 ± 26	u	89 ± 30	75 ± 27	49 ± 8	60 ± 14	72 ± 16
Intraburst frequency IM	73 ± 15	74 ± 22	81 ± 29	89 ± 25	64 ± 16	72 ± 20	u	84 ± 29	u	71 ± 19	81 ± 31	54 ± 13	58 ± 14	77 ± 25
CA1d SWRs (130–230 Hz)														
*n* SWRs	40	33	13	35	61	149	u	50	u	27	45	13	32	79
Mean rate inside (Hz)	21.71	47.44	u	15.16	0.65	9.97	u	6.2	u	1.73	10.27	u	24.78	15.61
Lambda rate outside (Hz)	53.81	61.54	u	26.84	21.82	36.66	u	15.83	u	24.45	15.16	u	8.19	31.51
SWR index	−0.43	−0.13	u	−0.28	−0.94	−0.57	u	−0.44	u	−0.87	−0.19	u	0.5	−0.34
Poisson *P*-value	<0.0001	0.01	u	0.005	<0.0001	<0.0001	u	0.003	u	<0.0001	0.05	u	<0.0001	0.001
Mean rate before ripple (Hz)	55.50	62.27	u	34.71	22.13	33.32	u	12.41	u	26.30	13.22	u	20.16	37.30
Mean rate after ripple (Hz)	59.63	65.00	u	50.00	30.08	46.81	u	46.11	u	36.85	23.22	u	21.25	45.53
Rebound index	0.04	0.02	u	0.18	0.15	0.17	u	0.58	u	0.17	0.27	u	0.03	0.10
P-value (KS-test)	0.09	0.54	u	0.002	0.002	<0.0001	u	<0.0001	u	0.13	<0.0001	u	0.14	<0.0001
CA1d theta (5–12 Hz)														
Theta cycles	419	292	448	220	30	429	u	374	u	217	229	165	120	517
Theta off %	0%	0%	0.9%	7,7%	0%	1,6%	u	14,9%	u	19,4%	13,1%	18%	1,7%	0,2%
LFP location	SP	SP	SP	SP	SP	SP	u	SP	u	SP/SR	SP	SP	SP	SP
Preferred theta phase	251°	272°	340°	290°	u	355°	u	234°	u	79°	320°	u	29°	342°
Mean vector length (*r*)	0.2	0.16	0.19	0.36	u	0.04	u	0.3	u	0.22	0.21	u	0.21	0.15
Rayleigh *P*-value	<0.0001	<0.0001	<0.0001	<0.0001	0.282	0.03	u	<0.0001	u	<0.0001	<0.0001	0.983	<0.0001	<0.0001
Total *n* spikes	2490	1785	2081	593	505	1674	u	1206	u	522	370	276	217	3943
Spikes per cycle	8.1	6.9	5.8	3.1	3.2	4.3	u	3.1	u	2.3	2.9	1.8	2.9	8.2

Positive (+) or undetectable (−) immunoreactivity to a molecule observed within subcellular domain.

Firing rates are expressed as mean of 1 s bins ± s.d. M, movement (locomotion and/or small movements). IM, immobility. Bursts are defined as >3 spikes with ISIs <40 ms or <20 ms (see burst incidence). Interburst intervals are in ms (median, interquartile range). Intraburst frequency expressed as mean ± SD (Hz). Some parameters for neurons TV78l and TV77q have been previously reported (Viney et al., 2018). Additional tests: The dendrites of TV78l and TV68a were immunopositive for SMI32.

*^a^*Soma was not recovered.

*^b^*Labeled soma and dendrites could not be directly linked to the projection axon.

*^c^*Firing patterns could not be unequivocally matched to the labeled neuron.

RSg, Granular retrosplenial cortex; PrSd, dorsal presubiculum; SUBd, dorsal subiculum; s, soma; n, nucleus; d, proximal dendrite; a, axon; t, axon terminals; x, tested but inconclusive; u, unknown.

##### Firing patterns.

Mean firing rates were calculated in 1 s windows within each behavioral state. Bursts were defined as a train of >3 spikes with interspike intervals (ISIs) of <20 ms and <40 ms. We included bursts with <20 ms ISIs to better capture the short-duration bursts of Teevra cells for comparison with LRNs and orchid cells. The <40 ms ISIs are also reported to be consistent with published data (Viney et al., 2018). Burst incidence was defined by the total number of bursts in 1 s windows. Interburst intervals were calculated by measuring the time elapsed between the first spikes of consecutive bursts (for <40 ms ISIs). Intraburst frequency was calculated from the number of spikes within the duration between the first and last spike of each burst (also for <40 ms ISIs).

##### Rhythmicity index (RI).

**Figure 1.**
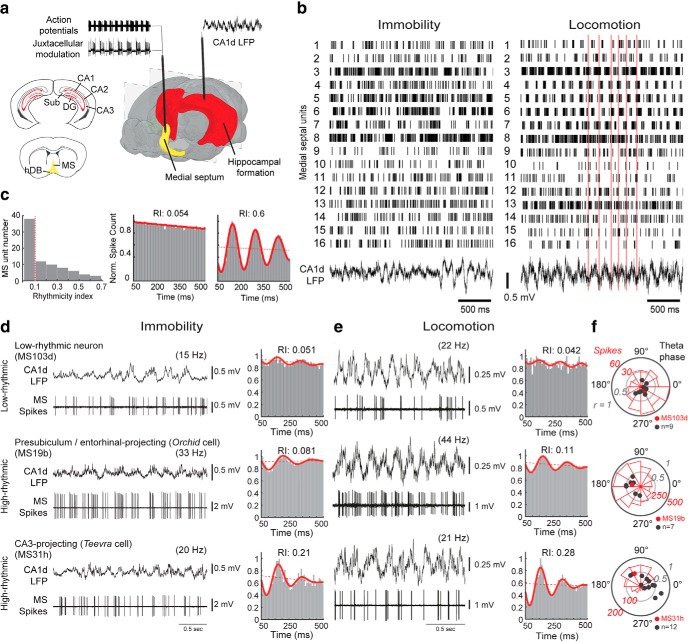
LRNs are distinct from HRNs recorded in awake mice. ***a***, Schematic of recording configurations. Action potentials of single medial septal neurons were recorded extracellularly using a glass electrode followed by juxtacellular labeling. Multiunit recordings were made using a silicon probe with 16 contacts in tetrode configuration (4 tetrodes) (Joshi et al., 2017). LFPs in CA1d, targeted to SP, were recorded in both configurations. Scheme is adapted from the Allen Institute Brain Explorer 2. Red indicates the hippocampal formation; yellow, MSDB. ***b***, Simultaneously recorded medial septal neurons (isolated units 1–16) display diverse firing patterns during immobility and locomotion on the jet ball. During locomotion, the mouse was running along a virtual linear track toward a reward (see Materials and Methods). Red bars are aligned to the troughs of some CA1d theta cycles. ***c***, Left, MSDB units (including those in ***b***) ordered by the RI. Vertical dashed line, RI cutoff of 0.1. Middle and right, Autocorrelograms of spikes during locomotion (50–500 ms, normalized counts) for two cells from ***b***. ***d***, ***e***, LRNs and HRNs recorded with glass electrodes during immobility and locomotion followed by juxtacellular labeling. Parentheses show mean firing rates. Top, An LRN increases firing frequency from immobility to locomotion. Middle, An entorhinal cortex-projecting orchid HRN (Viney et al., 2018) has long-duration bursts and increases firing from immobility to locomotion. Bottom, A CA3-projecting Teevra HRN (Joshi et al., 2017) has short-duration bursts and does not change its firing frequency from immobility to locomotion. Red bars are examples of bursts relative to CA1d theta. Autocorrelograms of spikes during immobility and locomotion reveal rhythmicity levels. ***f***, Polar plots of spike counts in 30° bins (red) for the 3 neurons from ***d*** and ***e*** relative to CA1d pyramidal layer theta (180° is peak). Radius is the vector length (*r*). Red points are the preferred mean theta phase for each example neuron. Black points are the preferred mean theta phase for other labeled neurons from each group (LRNs, *n* = 11; HRNs: orchid cells, n = 8; Teevra cells, *n* = 13). Sub, Subiculum; hDB, horizontal diagonal band.

The RI was calculated according as described previously (Joshi et al., 2017) and is based on the “theta index” (Royer et al., 2010). Briefly, data were prepared by calculating the spike time autocorrelogram (bin width 10 ms, maximum lag 500 ms) for spikes defined in periods of mobility or immobility. For acute silicon probe recording data, RI was calculated from time periods where the trained mouse had a consistent movement period on a virtual linear track. The autocorrelogram was normalized by dividing the peak value between 100 and 200 ms (range chosen to match the theta-frequency first side band) and center values were clipped so that the overall maximum was 1. We then fit a linear trend line to the above and performed a nonlinear fit (using the MATLAB lsqnonlin function) to the detrended data. The fitting function is a Gaussian-modulated cosine function with three parameters: (1) cosine (theta) frequency in Hz (between 4 and 8); (2) the peak value of the Gaussian scaling function (high value indicates strong short-latency theta modulation), and (3) SD (width) of Gaussian scaling function (high value indicates prolonged theta modulation). The solid red lines in Figure 1 are the fitted sinusoid functions (oscillatory frequency of the neuron) and the trends are shown by dotted lines. A coefficient of determination was measured at this stage to measure goodness of fit. After fitting, the rhythmicity index was calculated as follows: (1) for each peak and trough in 50–500 ms, the absolute value of the fitted sinusoid was divided by the corresponding trend line value (between zero and one) and (2) the rhythmicity index was taken as the mean of these trend-normalized peak values.

##### Firing probability during SWRs.

The power of the 130–230 Hz band-pass-filtered CA1d LFP was used to detect SWRs, with a threshold of at least 4 SDs above the mean power. We analyzed the firing probability of medial septal neurons during hippocampal SWRs as described previously (Katona et al., 2014; Unal et al., 2018). Neurons with fewer than 20 detected SWRs were excluded from analysis. Briefly, SWR-related firing rates have been compared with firing rates obtained outside of SWR events. Firing rates have been calculated for the *n* detected SWRs. Next, a population of 1000× *n* “surrogate SWR” time windows was generated sequentially. Surrogate SWRs were restricted to immobility periods when the majority of SWRs occurred. For each of the 1000 sets, individual firing rates were calculated for the *n* “surrogate SWRs” and their average was derived. Due to the limited recording periods, each spike was included in “surrogate SWRs” repeatedly, but in different time frames. The firing rates during detected SWRs were compared with the average rates during “surrogate SWR” periods using a two-sample Kolmogorov–Smirnov test. Finally, for each neuron, a SWR index was calculated with values between −1 (no firing during SWRs) and +1 (firing exclusively during SWRs), with 0 meaning no change in firing rate during SWRs compared with outside these events. Next, the rebound in SWR-related activity of low rhythmic single neurons was quantified by comparing the average firing rate 200 ms before the beginning and 200 ms after the end of *n* detected SWRs. For each SWR, a “rebound index” was calculated with values between −1 (only firing before and not after SWRs) and +1 (only firing after and not before SWRs), with 0 meaning no change in firing rate between before and after SWRs. To statistically test a rebound after SWR activity of individual single cells, a population of 1000× *n* “surrogate SWR” time windows were generated sequentially as was done for the mean firing rate comparison. For each of the 1000 sets, individual firing rates 200 ms before and 200 ms after the *n* surrogate SWRs were used to calculate rebound indices and their distributions were derived. The median distribution of the 1000× “surrogate SWR” rebound index distributions was compared with the measured distribution of rebound indexes using a Kolmogorov–Smirnov test as above.

#### Delineation of brain regions and neuronal reconstructions

Mouse brain atlas (Paxinos and Franklin, 2001) images of coronal brain sections reacted for acetylcholinesterase at 1.0–0.4 mm anterior–posterior (AP) relative to bregma were taken as a reference to identify the recording location of labeled and recovered cells within the medial septum. Based on the DAB and/or fluorescence immunoreactions, the location of somata was estimated for each brain and compared with the brain atlas based on the structure of the anterior commissure, ventricles, medial septum, and the extent of the horizontal diagonal band. The medial-lateral position of the anterior commissures in relation to the ventricles was a reliable estimate for the AP position.

Allen Brain Atlas images of selected marker genes expressed in coronal mouse brain sections were used as reference sections to define the positions of labeled LRN axons within processed fluorescence and DAB-reacted sections. Regional, subregional, and laminar boundaries were assigned based on gene expression profiles (see below) and a series of DAB-reacted coronal and sagittal sections immunoreacted for CB, PCP4, and RGS-14. We classified CA2 SR as including SL because this layer in CA2 is very thin compared with SL found in CA3. The alveus was included with SO because there is no discernible border or difference in the coverage by interneuron dendrites. Reconstruction of neuronal processes within each series of DAB-reacted sections were performed using Neurolucida, as described previously (Unal et al., 2018; Viney et al., 2018). The reconstructed axons were scaled up to the original unprocessed (freshly sectioned) *z*-thickness. Reconstructions of somata, dendrites, and local axon in the MSDB were performed on a drawing tube in 2D (Leitz Dialux 22 microscope). Soma and dendrites of TV78l were reconstructed in 2D by tracing over the fluorescence images on a computer monitor. Reconstructions are available at: http://neuromorpho.org/.

##### Defining subregions of the hippocampal formation by gene expression profiles.

We divided the hippocampal formation (DG, CA3, CA2, CA1, and SUB) along its septotemporal axis into a septal third (closest to the MSDB), a middle third, and a temporal third (farthest from the MSDB). We defined each region into subregions based on dorsal, intermediate, and ventral gene expression profiles (*Amigo2*, *Col15a1*, *Serpinf1*, *Wfs1*, and *Slc17a6 in situ* hybridization data from the Allen Institute). We analyzed combinations of specific gene expression patterns to define different subregions starting with previously published data (Fanselow and Dong, 2010). Because some genes show gradients along the septohippocampal axis, we selected genes that were the most representative for the dorsoventral axis, which correspond to functional domains of the hippocampus (Fanselow and Dong, 2010). Therefore, we divided CA3 into CA3d (closest to DG) and CA3i (closest to CA2) as their marker genes *Col15a1* (Collagen, type XV, α 1; http://mouse.brain-map.org/experiment/show/74511791) and *Serpinf1* [serine (or cysteine) peptidase inhibitor, clade F, member 1 (Sdf3); http://mouse.brain-map.org/experiment/show/2559] did not show any significant septotemporal gradient. The borders between CA2 and CA1d and between CA1d and dorsal subiculum (SUBd) were defined by *Wfs1* [Wolfram syndrome 1 homolog (Wolframin); http://mouse.brain-map.org/experiment/show/74881161] and *Amigo2* (adhesion molecule with Ig-like domain 2; http://mouse.brain-map.org/experiment/show/71250310) and *Wfs1* and *Slc17a6* [solute carrier family 17 member 6 (VGLUT2); http://mouse.brain-map.org/experiment/show/73818754], respectively. Unlike in rat, the radial extent of CA2 in mouse could not be reliably defined based on CB immunoreactivity, so we used a combination of PCP-4 and RGS-14 immunoreactivity when assigning varicosities to different subregions and layers. We defined CA1i as ventral of *Wfs1*-expressing CA1d, caudal of *Amigo2*-expressing CA2, and dorsal of the ventral subiculum (SUBv). The border of SUBv was defined as the region where stratum oriens (SO) of CA1i tapers away. Therefore, SUBi was defined as being ventral of SUBd caudal of CA1d *Wfs1*-expressing cells. The SUBi–SUBv border was defined as being parallel with the more rostral SUBv-CA1i border. Note that “dorsal” subregions can be found at all three septotemporal levels.

##### Determination of innervated cortical volumes.

The coordinates of varicosities entered in Neurolucida were imported into Mathematica (for the reconstruction of neuron TV68a). For the groups of varicosities assigned to each region (e.g., CA3, DG), outliers were identified by clustering (FindClusters) using the squared Euclidean distance function and a hierarchical clustering method (agglomerate, with weighted average linkage). A convex hull (ConvexHullMesh) was fitted around all varicosities assigned to each region with the detected outliers removed and the volumes of innervation for each region were recorded. The total innervated volume (sum of region volumes) and total number of varicosities (sum from all regions) were used to calculate the average varicosity density. A varicosity number for each region was calculated assuming a uniform distribution of varicosity occurrence in the innervated volume at the average density (average density multiplied by the region volume). The Pearson χ^2^ test was used to test the hypothesis that the observed numbers of varicosities distributed across regions did not differ from the numbers calculated from a uniform distribution.

#### Quantification of virally labeled axons and number of axon terminals

Virally labeled GFP-immunoreacted MSDB axons from VGAT^Cre^ and PV^Cre^ mice were visualized in the DG and CA3 regions and *z*-stacks were acquired from 70-μm-thick coronal sections at 10× magnification (typically 10–14 optical sections) using wide-field epifluorescence and Axiovision software. At least one image was acquired in both the DG and CA3 at three different septotemporal levels of the hippocampal formation and hemispheres were treated as independent. In Fiji (Schindelin et al., 2012), three rectangular regions of interest (ROIs) were created perpendicular to the layers, crossing all layers and avoiding the fimbria, with a width of 20 μm. Axons were counted if they crossed the entire width of the ROI. Counting was done blind to the mouse line. Because viral titer, injection volume, expression, and GFP immunofluorescence varied, ratios were calculated by taking the mean number of axons from the three ROIs in the DG divided by the mean number from CA3. An index was also calculated for each region as follows: [(∑*A*/*n*) − (∑*B*/*n*)/(∑*A*/*n*) + (∑*B*/*n*)], where *A* and *B* are the mean number of axons in the DG and CA3, respectively. Data were analyzed in Mathematica and Python.

To quantify the number of axon terminals in a given area from a neurobiotin-labeled cell, we used several methods. For Neurolucida reconstructions, we assigned axon terminals (varicosities) during the reconstructions and computed the totals. Parts of the reconstruction were validated independently by other researchers using light microscopy. We also counted terminals from epifluorescence *z*-stacks consisting of entire sections that contained strongly labeled axons. Parts of these *z*-stacks were validated by high-resolution confocal microscopic *z*-stacks. Finally, the drawing tube was used to document in 2D several DAB sections containing labeled axons. This was validated by at least two researchers independently drawing some of the same sections.

#### Statistics

For all methods, *p*-values and confidence intervals were calculated according to α = 0.05 and the analyses were performed using standard functions and custom-written code in MATLAB (Statistical Toolbox) and Mathematica (Wolfram Research). We have not estimated the minimal population sample for statistical power, but the number of animals and labeled neurons were similar to or larger than those used in previous works (Joshi et al., 2017; Viney et al., 2018). Nonparametric tests were applied to non-normally distributed data. Kruskal–Wallis one-way ANOVA or Mann–Whitney *U* test and two-sample Kolmogorov–Smirnov tests were used to compare between two groups and two distributions, respectively. Uniformity of circular distribution of spikes has been tested using Rayleigh's method (Zar, 1999). The mean depth of theta modulation and the preferential mean theta phase of firing of a given neuron have been computed using circular statistics. For the comparisons of firing phase preferences of different medial septal cell types, we used a two-sample permutation test (Good, 2000; Tukker et al., 2007).

## Results

### Cofiring of HRNs and LRNs

Medial septal neurons recorded in rats (Sweeney et al., 1992; King et al., 1998; Dragoi et al., 1999) and mice (Joshi et al., 2017) display varying degrees of rhythmicity in their firing patterns during locomotion and rest, which may differentially modulate the activity of their postsynaptic target neurons in the cortex and also locally in the MSDB. We encountered medial septal neurons that exhibited high or low rhythmic firing patterns in drug-free, head-restrained mice (Fig. 1). We simultaneously recorded multiple neurons using silicon probes in the MSDB of mice trained to run along a virtual linear track (*n* = 81 isolated units, *n* = 4 mice; Fig. 1*b*) (Joshi et al., 2017). Individual units showed various degrees of rhythmicity during both immobility and locomotion. Based on an RI during locomotion (Fig. 1*c*; Materials and Methods), we observed a large group of LRNs that we defined as having an RI < 0.1 [(*n* = 38, median RI: 0.04, interquartile range (IQR): 0.02–0.08]. We defined HRNs as having an RI ≥ 0.1 (*n* = 43, median RI: 0.32, IQR: 0.19–0.45), which include the Teevra and Komal clusters, as reported previously (Joshi et al., 2017). These data demonstrate that a large group of LRNs cofire with HRNs in mice across different behavioral states.

We hypothesized that LRNs project to cortical regions that are distinct from those innervated by the previously defined HRNs (Joshi et al., 2017; Viney et al., 2018) and/or LRNs have different postsynaptic targets from those of HRNs. Consistent with the multiunit recordings, we encountered LRNs intermingled with HRNs during single recording sessions when lowering a glass electrode through the MSDB as the mouse moved or rested on a circular treadmill (Fig. 1*a*,*d–f*). To reveal the identity of LRNs, we successfully recorded and labeled 12 LRNs [*n* = 10/12 with RI < 0.1 during movement; one neuron (MS68a) with RI = 0.14; one neuron (TV78l) with qualitatively low-rhythmic firing from a recording that lacked substantial movement; Table 2]. We included neurons MS68a and TV78l because they shared many features of labeled neurons with RI < 0.1.

**Figure 2.**
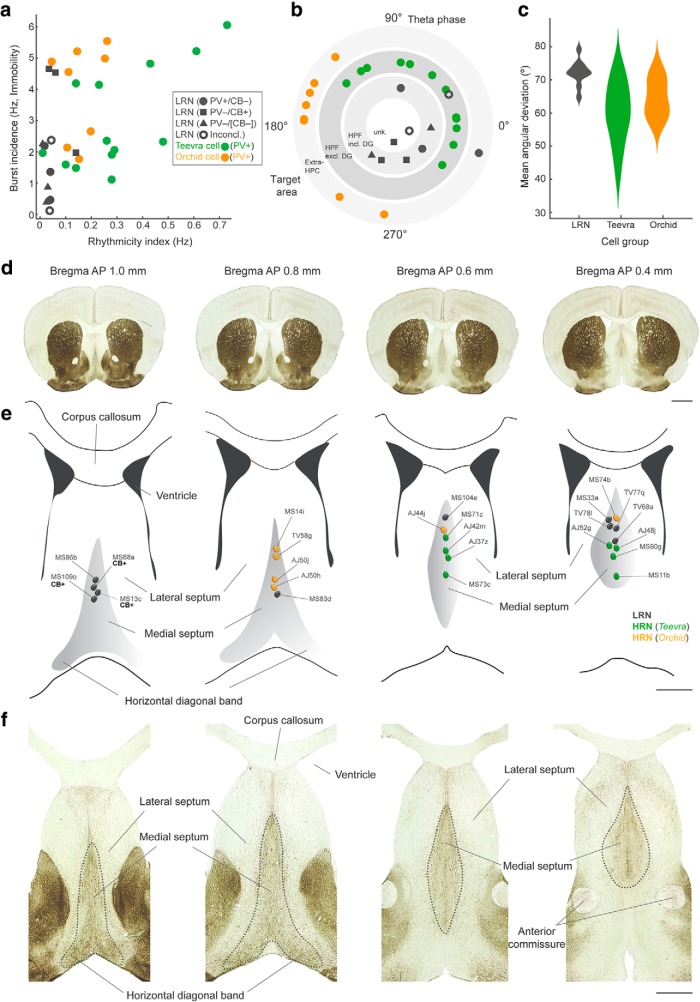
Comparison of LRN and HRN firing patterns and their recording locations. ***a***, Identified LRNs have a low burst incidence (20 ms bursts) and a low RI compared with identified HRNs (green, Teevra cells; orange, orchid cells). A combination of parameters including axonal target regions, molecular profiles, and firing patterns were used to define labeled (identified) LRNs and HRNs. One Teevra cell (MS11b) and one orchid cell (MS14i) was recorded in untrained mice, resulting in RI < 0.1 for these two neurons. ***b***, Polar plot of preferred mean theta phase relative to CA1d pyramidal layer theta (180° is peak) for labeled LRNs and HRNs ordered by target area(s): hippocampal formation (HPF) including DG; HPF excluding DG (i.e., cornu ammonis and subiculum); extra-HPF (presubiculum, parasubiculum, entorhinal cortex). Two had unknown target areas (see also Table 2). LRNs, *n* = 11; orchid cells, *n* = 8; Teevra cells, *n* = 13. ***c***, Distribution chart of mean angular deviation for the three groups. ***d***, Coronal mouse brain sections reacted for AChE at 1.0–0.4 mm AP relative to bregma (left to right). ***e***, Schematic coronal illustrations of the MSDB at four different AP positions according to ***d*** (left to right) showing the soma location of the LRNs and HRNs. The location of neuron MS13c (dashed line) is based on a proximal dendrite. ***f***, Coronal AChE sections of the MSDB at the same AP positions as in ***e*** (left to right). Coronal AChE images were taken from Paxinos and Franklin (2001). Scale bars (mm): ***d***, 1; ***e***, ***f***, 0.5. Inconcl., Inconclusive for PV and CB; Unk., unknown target region(s).

**Figure 3.**
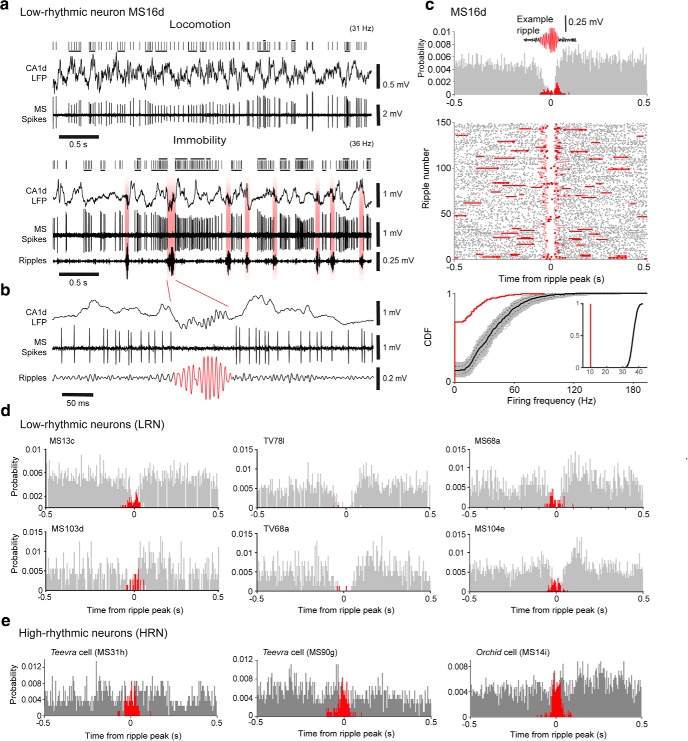
LRNs are suppressed during hippocampal sharp-wave ripples. ***a***, Activity of a SWR-suppressed LRN (MS16d) during locomotion (top) and immobility (bottom). Note absence of action potentials during SWRs (filtered 130–230 Hz, highlighted red). Black ticks indicate all detected spikes from the single neuron; top black bars are bursts of >3 spikes with ISIs <20 ms; bottom black bars are bursts of >3 spikes with ISIs <40 ms. Mean firing rates are shown in parentheses. ***b***, High time resolution of a ripple from ***a***. ***c***, Firing probability density (top) and raster plot (middle) relative to all detected SWRs (*n* = 149) during immobility for neuron MS16d. Continuous staggered lines in raster delineate start and end of aligned ripples; horizontal lines delineate adjacent ripples. Red indicates spikes during SWRs; gray indicates spikes outside of SWRs. Bottom, Comparison of mean firing rates during SWRs and periods outside of SWRs. The distribution of firing rates during individual SWRs (red) displayed as a cumulative distribution function (CDF) is shifted to the left from the distribution of a surrogate set of 1000 firing rate distributions during periods outside of SWRs (gray; median, solid black line; 95% confidence intervals, broken lines). Inset shows a comparison of mean SWR-related firing rate (red) with the distribution of surrogate mean SWR-related rates (black). ***d***, ***e***, Examples of the firing probability densities of other recorded and labeled LRNs (***d***) and HRNs (***e***) relative to SWRs during immobility.

**Figure 4.**
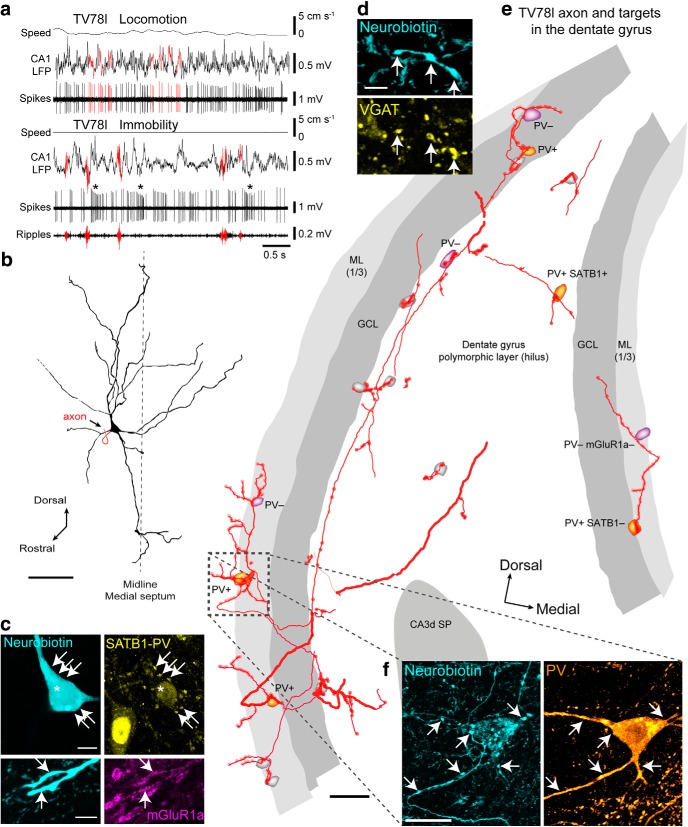
Firing patterns and synaptic targets of a GABAergic LRN mainly targeting the DG. ***a***, Top, Firing of TV78l during locomotion. Some spikes and their timing relative to theta oscillations (CA1 LFP) are highlighted in red. Bottom, During immobility, TV78l fired irregularly with accommodating bursts (asterisks). The cell did not fire during SWRs (red). ***b***, Reconstruction of the soma and dendrites in the MSDB. Arrow indicates the projection axon. Dashed line indicates the midline (dorsal is up). ***c***, Top, Soma of TV78l (cyan, neurobiotin, Nb, asterisk) was SATB1^+^ (yellow, nucleus) but lacked detectable cytoplasmic immunoreactivity for PV (also yellow). Some PV^+^ terminals were apposed to the soma and proximal dendrites (e.g., arrows). Note SATB1^+^/PV^+^ neuron at bottom left. Bottom, Dendrite (cyan, neurobiotin, arrows) was mGluR1a^+^ (magenta). ***d***, Terminals (cyan, neurobiotin) in the dentate gyrus were VGAT^+^ (yellow, arrows). ***e***, Partial reconstruction of the TV78l axon (red) from one 100-μm-thick section of temporal DG. Varicosities are highlighted in red. Some interneuron targets could be identified: orange, PV^+^; magenta, PV^−^; gray, untested. ***f***, A neuron with high mitochondrial biotin content was PV-immunoreactive (orange) in the ML (boxed region in ***e***) and received extensive basket-like innervation (>37 varicosities) from TV78l (cyan, neurobiotin). Arrows highlight some terminals on the soma and proximal dendrites. Many terminals overlap the soma due to the 2D projected *z*-stack. Images (*z*-thickness in μm and *z*-projection type): ***c***, top/bottom 3/4.2 maximum, ***d***, 4.1 maximum, ***f***, 9.5 SD. Scale bars (μm): ***b***, 100; ***c***, 10 and 5 (top and bottom); ***d***, 5; ***e***, 50; ***f***, 20.

**Figure 5.**
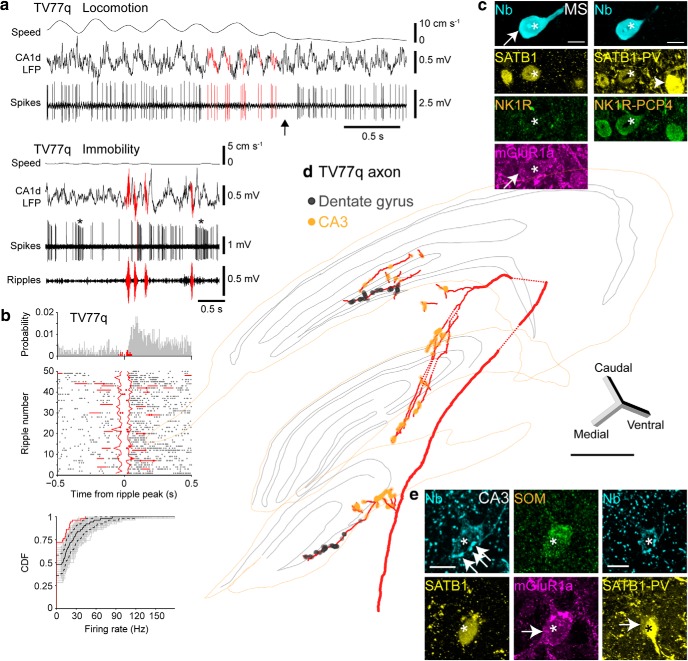
Firing patterns, cortical target regions, and target neurons of a LRN innervating both CA3 and the DG. ***a***, Firing patterns of LRN TV77q during locomotion (top) and immobility (bottom). Red indicates examples of the descending phases of theta cycles in the CA1 LFP and associated spikes (top) or SWRs (bottom). Arrow indicates theta skipping cycle. Note reduction in firing during SWRs. Asterisks indicate accommodating bursts. Note different time scales for the two behavioral states. ***b***, Firing probability density during SWRs (red), outside SWRs (gray), and spike raster (middle) relative to detected ripples. Continuous staggered lines in raster delineate start and end of aligned ripples; horizontal lines delineate adjacent ripples. Note rebound firing after ripples. Bottom, Cumulative distribution functions (CDFs) of firing rates during ripples for real (red) and 1000 shuffled (gray) distributions from immobility periods. Black curve is the median shuffled distribution; dashed, 95% confidence intervals. ***c***, TV77q soma (cyan, neurobiotin, asterisk) was SATB1^+^ (top, yellow) and mGluR1a^+^ (top, magenta, arrow) but NK1R^−^ (top, green). The cell was subsequently determined to be PCP4^+^ (bottom, green) and PV^−^ (bottom, yellow). Arrowhead indicates a nearby PV^+^ neuron. Note PV-immunoreactive terminals around the labeled cell. ***d***, Partial reconstruction of the axon (red) and varicosities in DG (gray) and CA3 (orange). Dashed lines indicate interpolated axon. ***e***, Terminals of TV77q (cyan, neurobiotin, arrows) were apposed to a soma containing a high level of endogenous biotin (asterisk) in CA3 SR, which was SOM^+^ (green), SATB1^+^ (left, yellow), and mGluR1a^+^ (magenta, arrow). The targeted cell was subsequently shown to be weakly PV^+^ (right, yellow, arrow). Images (*z*-thickness in μm and *z*-projection type): ***c***, left/right 3/0.77 maximum, ***e***, Left/right 8/8.4 maximum. Scale bars (μm): ***c***, ***e***, 10; ***d***, 500.

**Figure 6.**
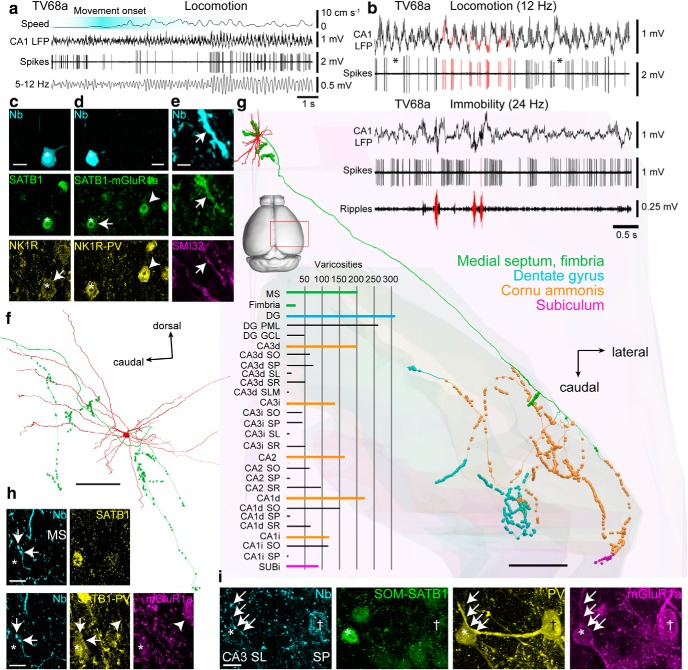
Firing patterns, cortical innervation, and targets of an identified LRN innervating multiple hippocampal areas. ***a***, Cell TV68a decreased firing during movement onset (top, highlighted cyan). Bottom, 5–12 Hz theta-filtered CA1d LFP. Note the absence of spikes for several theta cycles and an increase in rate with a higher theta amplitude. ***b***, The cell was coupled to the trough/ascending phase of theta oscillations during movement (top, examples in red) and was mostly silent during SWRs (bottom, red). Asterisks indicate theta skipping. ***c***, Soma (cyan, neurobiotin, asterisk) was SATB1^+^ (green, nucleus) and weakly NK1R^+^ (yellow, arrow, membrane). ***d***, Soma was subsequently determined to be mGluR1a^+^ (green, arrow) and PV^+^ (yellow). Arrowhead indicates neighboring SATB1^+^/mGluR1a^+^/PV^+^ neuron. ***e***, Dendrite (cyan, arrow) was mGluR1a^+^ (green) and SMI32^+^ (magenta). ***f***, Reconstruction of the soma and dendrites (red) and local axon with varicosities (green) in caudal MS. ***g***, Full reconstruction of the projection axon. Varicosities are color coded. Background displays some contours of the hippocampus. Red box highlights location in the brain. Bar chart displays number of varicosities in each region (colored) and corresponding subregions (black). ***h***, Two axon varicosities (cyan, neurobiotin, arrows) were in close apposition to a medial septal neuron (asterisk) that was SATB1^+^ (top, yellow) and PV^+^ (bottom, yellow, tested after SATB1) but mGluR1a^−^ (magenta). Arrowhead indicates a nontargeted neuron that was mGluR1a^+^/PV^−^. ***i***; Axon terminals (cyan, neurobiotin, arrows) in CA3d SL were apposed to PV^+^ dendrites (yellow) of a SATB1^+^/mGluR1a^+^ neuron (asterisk) and a nearby high endogenous-biotin-expressing mGluR1a^+^/SATB1^−^ neuron (†). Images (*z*-thickness in μm and *z*-projection type): ***c***, 2.2 maximum, ***d***, 0.33 single, ***e***, 3 maximum, ***h***, top/bottom 2.96/1.85 maximum, ***i***, 13.0 maximum. Scale bars (μm): ***c***, ***d***, ***h***, ***i***, 10; ***e***, 3; ***f***, 100; ***g***, 500.

Despite their low rhythmicity, most labeled LRNs coupled to CA1d theta oscillations (*n* = 10/12), preferentially firing on the descending phase (group circular mean phase ± circular SD, 319.2° ± 54.2°, group mean vector length = 0.20, *n* = 10 neurons; Figs. 1*e*,*f*, 2*b*, Tables 2, 3). Labeled HRNs preferentially coupled to the trough (Teevra cells) and peak (orchid cells) of CA1d theta oscillations (37.5° ± 53.5°, *n* = 13 identified Teevra cells; 178.6° ± 39.1°, *n* = 8 identified orchid cells; Figs. 1*f*, 2*b*) (Joshi et al., 2017; Viney et al., 2018). The group mean theta phase of LRNs was significantly different from HRNs (identified Teevra cells: *p* = 0.03; identified orchid cells: *p* = 0.04; permutation tests). Interestingly, LRNs showed variable firing patterns during theta oscillations (Figs. 1*e*, 3*a*, 4*a*, 5*a*, 6*a,b*), with 5/12 neurons not firing on 7.7–19.4% of theta cycles (“theta off”; Figs. 5*a*, 6*a,b*, Table 2). This included “theta skipping,” in which spikes are absent from single theta cycles during locomotion (Figs. 5*a*, 6*a,b*) (Jeffery et al., 1995; Brandon et al., 2013). Binning CA1d theta power into 4 levels revealed that theta skipping could occur at all power levels (*n* = 4/4 tested neurons; Fig. 6*a*,*b*). The variability of LRNs was also evident from the wide circular distribution of spikes around the mean preferred theta phase, measured as the mean angular deviation (circular SD). The mean angular deviation of LRNs was significantly different from HRNs (median, interquartile range, LRNs: 72°, 2.5°; Teevra cells: 59°, 13°; *U* = 115, *p* = 0.00173, *n* = 10 LRNs, *n* = 13 Teevra cells; median and interquartile range orchid cells: 64°, 10°; *U* = 68, *p* = 0.01455, *n* = 10 LRNs, *n* = 8 orchid cells; Mann–Whitney *U* tests; Fig. 2*c*). This suggests that LRNs fire with more variability during theta oscillations, which is reflected by their lower theta coupling (and RI) compared with HRNs (Fig. 1*f*, Table 3).

**Table 3.** Properties high- and low-rhythmic neurons

	HRNs (RI > 0.1)		LRNs (RI <0.1)
	*Teevra* cells	*Orchid* cells	
CA1d theta (5–12 Hz)			
*n*	13	8	10
Preferred theta phase	37.5° ± 53.5°	178.6° ± 39.1°	319.2° ± 54.2°
Group mean vector length (*r*)	0.39 ± 0.18	0.38 ± 0.10	0.20 ± 0.08
Firing patterns			
*n*	13	8	12
Firing rate M (Hz)	37.2 ± 13.5	55.2 ± 14.1	31.9 ± 14.1
Firing rate IM (Hz)	40.7 ± 15.8	39.8 ± 8.1	29.7 ± 15.3
Burst (40 ms) incidence M (Hz)	4.40 ± 1.56	4.98 ± 1.30	2.08 ± 0.88
Burst (20 ms) incidence M (Hz)	3.47 ± 1.99	3.97 ± 1.42	1.97 ± 1.73
Burst (40 ms) incidence IM (Hz)	3.56 ± 1.23	4.24 ± 0.74	2.14 ± 1.08
Burst (20 ms) incidence IM (Hz)	3.02 ± 1.57	5.37 ± 1.54	1.82 ± 1.45

Firing rates are expressed as group mean of 1 s bins ± SD. Bursts are defined as >3 spikes with ISIs <40 ms or <20 ms (see burst incidence) and expressed as group mean ± SD. Interburst intervals are in milliseconds (median, interquartile range) and are expressed as group mean ±SD.

M, Movement (locomotion and/or small movements); IM, immobility.

We also tested the coupling of LRNs to CA1d slow-gamma (32–39 Hz) and mid-gamma (55–80 Hz) oscillations (Lasztóczi and Klausberger, 2017), which occurred intermittently during both movement and immobility periods. Individual LRNs preferentially fired on the peak and descending phases of either slow-gamma cycles (*p* ≤ 0.045, *r* = 0.08–0.16, *n* = 4/12 neurons, Rayleigh tests) or mid-gamma cycles (*p* ≤ 0.018, *r* = 0.07–0.21, *n* = 5/12 neurons, Rayleigh tests). One LRN (MS16d) showed moderate phase coupling to both frequency ranges with the same mean phase preference (214°±75°). These data suggest, like some HRNs (Viney et al., 2018), LRNs can couple to different kinds of behaviorally contingent network oscillations.

Firing rates (mean ± SD, 31.9 ± 14.1 Hz during movement; 29.7 ± 15.3 Hz during immobility; *n* = 12 LRNs; Tables 2, 3) were distinct from “slow-firing” neurons reported in freely moving rats (mean firing rates <4 Hz; (Zhang et al., 2011), which include cholinergic neurons (Simon et al., 2006). One cholinergic neuron that we recorded and labeled in the vertical DB of a head-restrained mouse (neuron TV43w, mean firing rates: 4.01 Hz during movement, 1.04 Hz during immobility) was consistent with slow-firing neurons and other basal forebrain cholinergic neurons recorded in awake mice (Hangya et al., 2015). In fact, mean firing rates of LRNs were similar to HRNs during immobility (Teevra cells: 40.7 ± 15.8 Hz, t_23_ = 1.8, *p* = 0.09, *n* = 13; orchid cells: 39.8 ± 8.1 Hz during immobility, *t*_(19)_ = 1.7, *p* = 0.11; *n* = 8; unpaired *t t*ests) and to Teevra HRNs during movement (Teevra 37.2 ± 13.5 Hz during movement, *t*_(23)_ = 0.97, *p* = 0.34; orchid cells: 55.2 ± 14.1 Hz, *t*_(19)_ = 3.6, *p* = 0.002, *n* = 8; unpaired *t* tests; Table 3). During locomotion, mean firing rates at low speeds (up to 3 cm s^−1^) were similar to rates at slightly higher speeds (3–6 cm s^−1^; *n* = 3/4 tested LRNs; MS13c, MS16d, TV77q). Only 1 of 4 tested LRNs showed an increase (18 Hz for <3 cm s^−1^ to 36 Hz for 3–6 cm s^−1^; MS33a). At higher speeds, 1 LRN showed a slight increase in its mean firing rate (37 Hz for 3–6 cm s^−1^ to 46 Hz for 6–9 cm s^−1^; TV77q).

### LRNs are suppressed during SWRs

Hippocampal SWRs occurred during immobility. These “awake SWRs” are implicated in memory consolidation (O'Neill et al., 2006; Carr et al., 2011) and the activity of many MSDB neurons is modulated during SWR events, including some HRNs (Dragoi et al., 1999; Borhegyi et al., 2004; Viney et al., 2013; Unal et al., 2018; Viney et al., 2018). We observed that LRNs decreased their firing during CA1d SWRs (*n* = 9/10 labeled LRNs with >20 recorded SWRs; firing rate distributions significantly different from a Poisson distribution, *p* < 0.05; Figs 3*a–d*, 4*a*, 5*a,b*, 6*b*, Table 2), contributing to their negative SWR indices (Table 2). They fired occasional accommodating bursts outside of SWRs (*n* = 9/10 LRNs; Figs. 4*a*, 5*a*) and six displayed significant “rebound firing” in the ∼250 ms immediately after SWRs (Figs 3*c*, 5*b*, Table 2; 2-sample Kolmogorov–Smirnov tests, *p* ≤ 0.002, *n* = 6). Most labeled HRNs did not significantly reduce their firing during SWRs (*n* = 5/5 tested Teevra cells; *n* = 4/5 tested orchid cells; see Fig. 3*e* and Joshi et al., 2017; Viney et al., 2018). This suggests that suppression during SWRs is a major but not exclusive characteristic of LRNs.

Outside of SWRs, LRNs exhibited some bursting activity, but the burst incidence was low (mean ± SD. burst incidence during immobility periods = 2.14 ± 1.08 Hz for bursts with <40 ms ISIs; 1.97 ± 1.73 Hz for <20 ms ISIs; *n* = 12 neurons, Figs. 1*d*, 2*a*, 3*a*, Tables 2, 3). This is in contrast to HRNs (King et al., 1998; Dragoi et al., 1999; Borhegyi et al., 2004; Joshi et al., 2017; Viney et al., 2018), which maintained a high burst incidence across behavioral states (orchid cells: 4.98 ± 1.30 Hz for <40 ms ISIs, movement periods; 4.24 ± 0.74 Hz for <40 ms ISIs, immobility; 5.37 ± 1.54 Hz for <20 ms ISIs, movement periods; 3.97 ± 1.42 Hz for <20 ms ISIs, immobility; *n* = 8; Teevra cells: 4.40 ± 1.56 Hz for <40 ms ISIs, movement periods; 3.56 ± 1.23 Hz for <40 ms ISIs, immobility; 3.47 ± 1.99 Hz for <20 ms ISIs, movement periods; 3.02 ± 1.57 Hz for <20 ms ISIs, immobility; *n* = 13, Table 3), contributing to a high RI (Figs. 1*c–e*, 2*a*). Conversely, LRNs had a low burst incidence during movement periods (2.08 ± 0.88 Hz for <40 ms ISIs; 1.82 ± 1.45 Hz for <20 ms ISIs; *n* = 12 neurons; Figs. 1*e*, 2*a*, 3*a*, Tables 2, 3). Next, we investigated how the SWR suppression, low burst incidence, and low rhythmicity were related to the cortical target regions of this subpopulation of MSDB neurons.

### Cortical target regions of septohippocampal LRNs

The LRNs projected septotemporally from the medial septum via the fimbria (*n* = 11/12; *n* = 1/12 via the fornix) and innervated at least one region of the hippocampal formation (*n* = 12/12). The majority branched within the septal one-third of the hippocampus along the septotemporal axis (i.e., closest to the MSDB) in CA3 (*n* = 6/8 neurons with sufficiently labeled axon; Table 2). One continued directly to the DG (MS13c, with collaterals occupying the molecular layer of both DG blades). Another LRN (MS104e) shared projection regions with orchid cells (Viney et al., 2018) via the dorsal fornix except that the axon did not reach the entorhinal cortex. Instead, terminals were observed primarily in the granular retrosplenial cortex (RSg) and CA1. Of the remaining four LRNs, axons in the fimbria were too weakly labeled to recover branches, except for one with partial axon observed in the DG (MS16d). Unlike CA3-projecting HRNs, LRNs that branched in CA3 continued and additionally branched in the DG (*n* = 5/6 neurons) or CA1d and the SUBd (*n* = 1/6; MS33a). We also labeled two other neurons that innervated both CA3 and the DG (MS09_ and MS53_), but recordings could not be assigned due to more than one labeled neuron being recovered (Table 2). Overall, we labeled 14 neurons that differed in their axon termination patterns from exclusively CA3-innervating HRNs (Teevra cells) and entorhinal-cortex-projecting HRNs (orchid cells) (Fig. 2*a*,*b*). Nine LRNs had collaterals in the DG, which receives the densest MS innervation (Figs. 4, 5, 6, Table 2).

**Figure 7.**
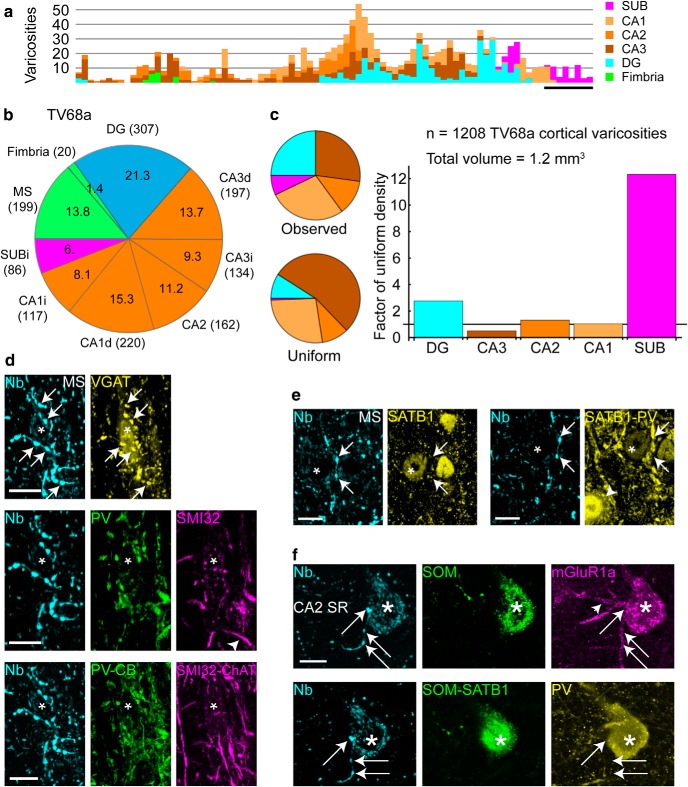
Distribution of varicosities and target neurons of a LRN innervating multiple hippocampal areas. ***a***, Number of TV68a cortical varicosities as a function of distance along the rostrocaudal axis color-coded by region. ***b***, Pie chart showing the proportions (%) of all varicosities, including those in the MS, in the innervated regions (parentheses: number of varicosities). ***c***, Left, Pie charts of varicosity number proportions by region: observed cortical varicosities and varicosity numbers were calculated from a uniform distribution at the overall average density (color-coded as in ***a***). Right, Bar chart of observed over calculated uniform proportions. Right, Bar chart of real over expected proportions. Horizontal line indicates level of no difference from expected. ***d***, A target neuron in the MS had several terminals of TV68a (cyan, neurobiotin, arrows) in close apposition to its soma (asterisk). Top, Terminals were immunoreactive for VGAT (yellow, e.g., arrows). Middle, The target neuron lacked detectable immunoreactivity for PV (green) and SMI32 (magenta). Arrowhead indicates SMI32^+^ dendrite (not targeted). Bottom, The target was immunonegative for CB (green) and ChAT (magenta); ChAT^+^ processes are present. ***e***, Another target neuron in the MS (asterisk) in close apposition to TV68a terminals (cyan, neurobiotin, arrows). The target was immunopositive for SATB1 (left, yellow, nucleus) and immunonegative for PV (right, yellow). Several PV-immunoreactive terminals were also in close apposition to the soma. Arrowhead, Nontargeted PV^+^ cell. ***f***, Terminals (cyan, neurobiotin, arrows) in CA2 SR were in close apposition to mGluR1a-immunoreactive dendrites (magenta) and soma (magenta, asterisk). The varicosity apposed to the soma was located at the crossing point of an mGluR1a-immunopositive dendrite from another neuron (arrowhead) and the vertical mGluR1a-immunoreactive dendrite also from another neuron. The soma was also immunoreactive for SOM and SATB1 (green, SATB1 tested after SOM) and PV (yellow). Images (*z*-thickness in μm and *z*-projection type): ***d***, top/middle/bottom 5.04/5.85/6.66 maximum, ***e***, top/bottom 2.96/2.96 maximum, ***f***, top/bottom 5.73/1.91 maximum. Scale bars (μm): ***a***, 200; ***d***, ***e***, 10; ***f***, 5. MS, Medial septum.

Of the neurons with axon observed in the DG, two had >90% of terminals distributed in the septal/middle two-thirds or temporal one-third of the DG across the septotemporal axis (MS68a and TV78l, respectively), with the remaining terminals in CA3. Neuron MS13c had terminals restricted to the septal one-third of the DG. Some labeled neurons innervated the DG with lower proportions (62% in DG vs CA3, MS09_; 30% in DG vs CA3, TV77q; Fig. 5). Strikingly, 2/9 neurons (TV68a, MS53_) exhibited a multiple-branching pattern originating in the fimbria within the septal one-third of the hippocampus, innervating restricted portions of the DG (∼20% of terminals), CA3, CA2, CA1, SUBd, and locally within the MSDB (Figs. 6, 7). The axonal proportions of two neurons were undetermined due to weak labeling, but they formed terminals in the middle septotemporal third of the DG (MS16d and MS103d). We conclude that LRNs innervate select regions of the hippocampal formation, with a bias toward the DG and CA3, and are distinct from the projections of HRNs such as Teevra and orchid cells (Fig. 2*b*). The combination of firing suppression during SWRs, a low burst incidence during immobility, and low rhythmicity during locomotion yielded a strong prediction of their axonal innervation of the cortex (Fig. 2*a–c*).

### Molecular profiles of LRNs

Next, we investigated how the molecular expression profiles of the 14 labeled neurons related to their target regions. We found that most neurons lacked detectable immunoreactivity for PV (*n* = 9/14 tested neurons PV^−^; 2 inconclusive; Figs. 2*a*,*b*, 4*c*, 5*c*, Table 2). Two PV^−^ neurons were innervated by PV^+^ terminals in the MSDB (Figs. 4*c*, 5*c*); these neurons were also immunopositive for Purkinje protein 4 (PCP4, Table 2). Of the 3 PV^+^ neurons, one innervated multiple regions that included the DG and CA3 (TV68a; Fig. 6*c*,*d*). The second (MS103d) was partially labeled and small axonal branches were observed in CA3 before the DG. The PV^+^ neuron MS104e did not innervate the DG. Only a minority of labeled neurons were CB^+^ (*n* = 3/14 tested neurons, 3 inconclusive tests, Table 2), two of which showed strong preferential innervation of the DG (MS13c and MS68a). The axonal branching patterns of the third CB^+^ neuron (MS109o) could not be determined. None of the neurons was double immunopositive for PV and CB. Interestingly, the CB^+^ neurons were located in the most rostral part of the MSDB (Fig. 2*d–f*). Other labeled LRN somata were intermingled with labeled HRNs along the midline (Fig. 2*d–f*). We tested the terminals of eight neurons for the vesicular GABA transporter (VGAT), a marker for GABAergic neurons, and five were immunopositive (three PV^−^/CB^−^, three PV^+^, three inconclusive). Most neurons were also immunopositive for mGluR1a (*n* = 7/11 tested, 3 inconclusive) and the transcription factor SATB1 (*n* = 4/9 tested neurons, 2 inconclusive) (Figs. 4*c*, 5*b*, 6*c–e*, Table 2). We conclude that LRNs comprise a diverse subpopulation of MSDB neurons based on their molecular profiles, with at least three identifiable subgroups that send axonal termination to the DG: (1) PV^−^/CB^−^ GABAergic neurons that innervate different proportions of CA3 and the DG; (2) primarily DG-projecting CB^+^ neurons; and (3) mainly PV^+^ GABAergic neurons that innervate multiple regions that include CA3 and the DG (Fig. 2).

Three LRNs had similar firing patterns (MS13c, MS109o, and MS83d). We predict this group to be CB^+^/mGluR1^+^ LRNs (CB was tested but inconclusive for MS83d). These LRNs did not skip theta cycles, were coupled to the late descending/trough phase of theta, had a low RI, and exhibited a higher firing rate during movement than PV^−^/CB^−^ LRNs (Fig. 2*a*,*b*, Table 2). Neuron MS68a, another CB^+^ LRN, differed in its firing patterns from the others, having a high SWR rebound index (Fig. 3*d*, Table 2) and not firing on all theta cycles. It also lacked detectable immunoreactivity for mGluR1a, suggesting that it is part of a separate group of CB^+^ septohippocampal neurons. Interestingly, we observed CB^+^/GFP^+^ neurons along the midline of the MSDB in a VGAT^Cre^ mouse injected with a Cre-dependent AAV-expressing EYFP (data not shown) in the region where we recovered CB^+^ neurons (Fig. 2*d–f*). This suggests that different kinds of CB^+^ GABAergic MSDB neurons exist, some of which may target the hippocampal formation, in addition to those that target the entorhinal cortex (Fuchs et al., 2016).

### DG receives less PV^+^ septal innervation than CA3

**Figure 8.**
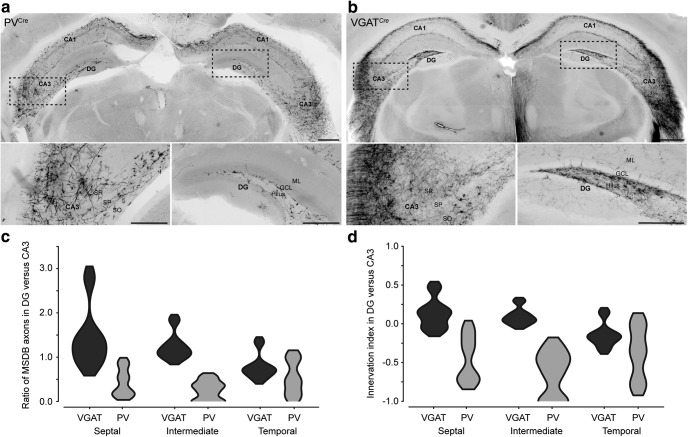
Differential innervation of the DG and CA3 by GABAergic and PV^+^ MSDB axons. ***a***,***b***, Coronal section from a PV^Cre^ mouse (***a***) and a VGAT^Cre^ mouse (***b***) showing MSDB innervation of the hippocampal formation (GFP, black). Bottom, MSDB axons in CA3 and DG, respectively, from boxed regions. ***c***, DG receives less PV^+^ innervation than CA3. Distribution chart of the ratio of MSDB axons in the DG versus CA3 in VGAT^Cre^ mice (GABAergic neurons, black) and PV^Cre^ mice (PV^+^ neurons, gray). ***d***, Distribution chart as in ***c*** but plotted as an index, where −1 and +1 represent a bias toward CA3 and the DG, respectively (see Materials and Methods). Images are wide-field epifluorescence montages, reverse contrast, 0.65 μm thick, at 10× magnification. Scale bars: ***a***, ***b***, top, 500 μm; bottom, 250 μm.

Based on the projections of PV^−^ LRNs and the lack of DG innervation by PV^+^ Teevra cells (Fig. 2*b*), we hypothesized that the DG receives less PV^+^ GABAergic input than CA3. To test this, we analyzed the proportion of PV^+^ axonal branches from the MSDB in the DG compared with CA3 (see Materials and Methods) in PV^Cre^ mice (*n* = 4 mice). We observed a low ratio of PV^+^ MSDB axons in the DG versus CA3 (median, IQR: septal third, 0.36, 0.54; middle third, 0.27, 0.40; temporal third, 0.47, 0.66; *n* = 548 axons, 13 regions, 4 mice; Fig. 8*a*,*c*). This low ratio could not be explained by the distribution of MSDB GABAergic afferents in the DG. We observed a high ratio of GABAergic MSDB axons in the DG versus CA3 at three septotemporal levels (septal, 1.28, 0.55; middle, 1.14, 0.25; temporal, 0.72, 0.17; *n* = 5216 axons, 18 regions, 3 mice; Fig. 8*b*,*c*). Overall, there was significantly lower ratio of PV^+^ MSDB axons in the DG versus CA3 compared with all GABAergic MSDB axons (*p* = 0.000057, *U* = 16, Mann–Whitney test; *n* = 13 and 18 regions from PV^Cre^ and VGAT^Cre^ mice, respectively; Fig. 8*c*,*d*). These data demonstrate that PV^+^ MSDB neurons represent a minority of the GABAergic MSDB input to the DG (Bao et al., 2017; Unal et al., 2018).

### Postsynaptic targets of low rhythmic PV^−^/CB^−^ septohippocampal neurons

We further investigated PV^−^/CB^−^ septohippocampal neurons by analyzing the distribution of their terminals and postsynaptic targets across CA3 and the DG (Table 2, 4, 5). The axon of LRN TV78l formed a single collateral in the intermediate CA3 (CA3i, see Materials and Methods for definitions) innervating the proximal dendrites of a PV^+^/SATB1^+^/somatostatin (SOM)^+^ interneuron in SO (*n* = 7 varicosities, Table 4), a marker combination that matches dendrite-targeting oriens lacunosum moleculare (OLM) cells and bistratified cells. Interneurons in CA3–CA1 that are PV^+^/SATB1^+^/SOM^−^ and PV^+^/SATB1^−^/SOM^−^ are basket cells and axo-axonic cells, respectively (Viney et al., 2013). The main axon in the fimbria continued to the temporal DG, where multiple terminals decorated the dendrites and somata of DG interneurons (Fig. 4*e*,*f*). Postsynaptic targets were in the polymorphic layer (PML, hilus), granule cell layer (GCL), and molecular layer (ML) (*n* = 815 sampled varicosities, 56% in the ML; Fig. 4*e*, Table 2). Most targets were PV^+^ (*n* = 10/17 tested targets; Fig. 4*e*,*f*, Table 4). By far the most extensively innervated targets were large interneurons at the GCL/ML border expressing high levels of endogenous biotin (Fig. 4*f*), which resembled axo-axonic cells (Soriano et al., 1990). In the PML, axons were observed along the GCL border, where they either targeted somata and dendrites of radially oriented neurons or branched superficially into the main hilar region, forming terminals on other neurons (Fig. 4*e*).

**Table 4.** Cortical target neurons of LRN TV78l

Target ID	Location	PV	SATB1	mGluR1a	Endobiotin	CR
S31A	CA3i SO	+	+	u	+	u
S31B		+	u	u	+	u
S21A	DG ML	u	u	u	−	u
S20B		u	u	u	+	u
S17A		+	u	u	+	u
S17B		+	u	u	+	u
S17C		−	u	u	u	u
S17D		−	u	u	u	−
S17G		−	u	u	u	−
S17K		−	u	u	u	−
S17L		+	−	−	u	u
S17M		−	u	−	u	−
S17N		+	u	u	u	u
S17O		+	u	u	u	u
S17P		−	u	u	u	−
S22A		u	u	u	−	−
S22B		u	u	u	−	−
S22C		u	u	u	+	−
S21B	DG ML/GCL	u	u	u	−	u
S17J		+	−	u	+	u
S16A		+	u	u	+	u
S21C	DG GCL	u	u	u	+	u
S17H		+	u	u	u	u
S21F	DG GCL/PML	u	u	u	+	u
S20A		u	u	u	+	u
S17F		+	u	u	+	u
S21D	DG PML	u	u	u	+	u
S21E		u	u	u	−	u
S17E		−	u	u	(+)	−
S17I		+	−	u	+	u
Total +		12/19	1/4	0/2	15/20	0/9

Molecular profiles of presumed postsynaptic neurons based on close apposition of axon terminals. Additional tests: targets S20A and S20B were also M2R^−^; S31A was SOM^+^; S22A and S22B were nNOS^+^ and S22C was nNOS^−^.

+, Detectable positive immunoreactivity or signal; −, undetectable immunoreactivity or signal in vicinity of immunopositive signals; u, unknown (unavailable or inconclusive). Parentheses indicate weak immunoreactivity or signal. Endobiotin, high levels of endogenous intracellular biotin.

**Table 5.** Cortical target neurons of LRN TV77q

Target ID	Location	Domain	PV	SATB1	mGluR1a	Endobiotin
S53A1*^a^*	CA3 SO	s	(+)	−	u	+
S53A2*^a^*		d	+	u	+	u
S53B1*^b^*		s	(+)	+	+	+
S53B2*^b^*		d	+	u	+	u
S53C		d	+	u	+	u
S53D		d	+	u	+	u
S53E		d	+	u	+	u
S53F		d	+	u	+	u
S53G		d	+	u	+	u
S53H		d	−	u	+	u
S53I		d	+	u	+	u
S53J		d	+	u	+	u
S53K		d	+	+	+	(+)
S53L		d	+	u	+	u
S52D		s	(+)	+	(+)	u
S52A	CA3 SO /DG PML	ds	+	+	+	(+)
S50A	CA3 SP	ds	+	−	+	+
S50B		d	+	u	+	u
S50C		d	+	u	u	u
S48A	CA3d SR/SLM	ds	+	+	+	+
S52B	DG PML	d	(+)	+	(+)	(+)
S52C		d	(+)	+	+	(+)
S52E		d	+	u	u	u
S47B		d	+	u	u	+
S44A		d	u	−	u	(+)
S49A	DG PML/GCL	ds	(+)	u	u	u
Total +			22/23 (24/25)	7/10	18/18 (20/20)	10/10

Molecular profiles of presumed postsynaptic neurons, based on close apposition of axon terminals.

+, Detectable positive immunoreactivity or signal; −, undetectable immunoreactivity or signal in vicinity of immunopositive signals; u, unknown (unavailable or inconclusive). Parentheses indicate weak immunoreactivity or signal. Domain, target subcellular domain (s, soma; d, dendrite).

*^a,b^*MSDB terminals were apposed to one or both of these targets in both cases and the targets themselves were in apparent contact; this affects the totals. Because many targets were onto thin distal dendrites, we cannot rule out that in some cases the target dendrites originated from the same neuron. Target S48A was also SOM^+^ and CB^−^.

In contrast to the intense and specialized cortical innervation of LRN TV78l, other PV^−^/CB^−^ septohippocampal neurons (Table 2) provided sparse innervation of interneurons, mostly on dendrites and rarely on cell bodies (*n* = 4/5 neurons), as was the case for PV^+^/CB^−^ LRNs (below). The PV^−^/CB^−^ neuron TV77q initially branched in the septal third of the hippocampus, where it formed terminals in CA3i and the DG PML and branched again across the middle third of the septotemporal axis innervating CA3i and CA3d, followed by the DG PML (Fig. 5, TV77q). In CA3 (*n* = 252/341 sampled varicosities), spiny PV^+^/mGluR1a^+^ interneurons were innervated (*n* = 17/20 tested dendrites and/or somata) and most of those tested were SATB1^+^ (*n* = 5/6, including a PV^+^/mGluR1a^+^/SATB1^+^/SOM^+^ neuron; Fig. 5*e*, Table 5). In two cases the same axon terminals were in close apposition to crossing points of two different dendrites from two neurons (Table 5), likely to be the site of a gap junction (Tamás et al., 2000; Baude et al., 2007). Within the DG (*n* = 89/341 sampled varicosities; 87 in the PML), terminals were apposed to PV^+^ neurons (*n* = 5/5 tested dendrites and/or somata), two of which were additionally SATB1^+^/mGluR1a^+^ (Table 5). Therefore, the main targets of PV^−^/CB^−^ LRN TV77q were PV^+^/mGluR1a^+^ neurons in CA3.

Similar to LRNs TV78l and TV77q, MS09_ branched in the fimbria close to CA3, with the other collateral continuing along CA3 SO to the DG. Along the septotemporal axis, terminals were observed in CA3i and CA3d within the middle third of the hippocampus and in the DG across the middle and temporal thirds. In the DG, the majority of terminals were in the PML (84% PML, 16% GCL); in CA3, most were in SP (50% SP, 43% SR). Like TV77q, MS09_ mostly formed sparse terminals along dendrites and rarely on cell bodies. We identified PV^−^ targets in both CA3 and the DG (*n* = 5/5 targets). One target was SATB1^−^/CR^−^, which expressed high levels of endogenous biotin and was located in CA3 SP; two neurons in CA3 SO were SATB1^−^/SOM^−^/CCK^−^; a SOM^−^/CCK^−^ neuron (SATB1 inconclusive) was located in the PML (17 terminals; 9 on the soma) and a SATB1^+^/M2R^−^ neuron (SOM inconclusive) was identified, also in the PML (7 terminals). Neuron MS16d had similar firing patterns to the other PV^−^/CB^−^ LRNs but was too weakly labeled to visualize targets.

### Projections of LRNs innervating multiple hippocampal areas

Three labeled neurons (TV68a, MS53_, MS103d) shared several features. We fully reconstructed GABAergic PV^+^/CB^−^ LRN TV68a (which was coupled to the ascending phase of theta oscillations and skipped theta cycles; Figs. 2*b*, 6*a*,*b*, Table 2), revealing a major innervation of the DG (Figs. 6, 7, Movie 1). To our knowledge, this is the first complete reconstruction of a recorded and labeled mouse basal forebrain neuron. Its dendrites extended rostrally in the MS and caudally into the septofimbrial nucleus (SFi) (Fig. 6*f*). Axonal varicosities were confined to the dorsocaudal MS (*n* = 199 septal varicosities; Figs. 6*f*, 7*b*) and in restricted subregions across the hippocampal formation (*n* = 1243 cortical varicosities; Figs. 6*g*, 7*a*,*b*). The observed distribution of TV68a cortical varicosities was significantly different from uniform (χ^2^ = 1400, *p* < 0.001; Fig. 7*c*) across the innervated subvolumes of each region, the DG, CA3, CA2, CA1, and SUB (1208 cortical varicosities within 1.2 mm^3^, outliers removed). A small volume of the SUB received a much greater innervation than if the varicosities were uniformly distributed (86 varicosities within 0.007 mm^3^), followed by the much larger DG volume (302 varicosities within 0.108 mm^3^). The CA1 and CA2 regions were innervated close to a uniform distribution, whereas CA3 received less innervation (Fig. 7*b*,*c*). In the DG, the PML was strongly innervated and the dorsal CA3 and CA1 received less input (Fig. 6*g*), followed by CA2 and CA3i. The majority of CA1-innervating terminals were located in SO. In contrast, strata pyramidale of CA1, CA2, and CA3i received very few terminals, with the exception of part of CA3d, which is closest to the DG (Fig. 6*g*). Therefore, analogous to the organization of some thalamocortical neurons (Clascá et al., 2016), there are select basal forebrain projections that innervate multiple, interconnected cortical regions.

Movie 1.Reconstruction of LRN TV68a. Green arrow indicates dorsal; red arrow, lateral; blue arrow, caudal. Movie is related to Figures 6 and 7.10.1523/JNEUROSCI.3024-18.2019.video.1
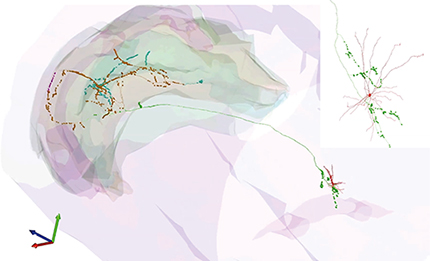

The sparse terminals of PV^+^ LRN TV68a targeted mostly PV^+^ neurons (*n* = 10/13) in CA2 and CA3, including a SATB1^+^ subset (*n* = 4/6, Table 6). Similar to LRN TV77q, some axon terminals were located at the crossing points of dendrites originating from two to three individual cells (Figs. 6*i*, 7*f*, Table 6), prime locations for gap junctions between interneurons (Tamás et al., 2000; Baude et al., 2007). The tested targets involved in such crossings in CA3 lacked detectable immunoreactivity for SOM, but showed differential immunoreactivity for SATB1 (Fig. 6*i*), suggesting that they were PV^+^ basket and axo-axonic cells (Viney et al., 2013). In the MS and SFi, the extensive local axon of TV68a targeted both PV^+^ (*n* = 6/9) and PV^−^ (*n* = 3/9) neurons (Figs. 6*h*, 7*d*,*e*, Table 6). The PV^−^ targets were innervated by PV^+^ terminals and 2/2 tested were SATB1^+^/mGluR1a^+^ (Fig. 7*e*), matching the target profile of other LRNs (Figs. 4*c*, 5*c*, Table 2). A different target was PV^+^/SATB1^+^/mGluR1a^+^ (Table 6), matching the target profile of TV68a and MS104e. No terminals were observed in extrahippocampal regions. The drop in firing rate of TV68a at the onset of movement suggests that these cortical and subcortical targets are simultaneously disinhibited during this behavioral state transition (Fig. 6*a*).

**Table 6.** Cortical target neurons of LRN TV68a

Target ID	Location	Domain	PV	SATB1	mGluR1a	Endobiotin	PV input	SOM
S76A	MS	d	+	u	−	−	u	u
S76B		s	−	+	+	−	+	u
S76C		ds	−	+	+	−	+	u
S76D		s	(+)	+	u	−	(+)	u
S76E		s	+	+	u	−	(+)	u
S76F		d	u	u	+	u	u	u
S76G		s	+	+	+	−	u	u
S76H		s	(+)	+	u	−	(+)	u
S74A		ds	−	u	u	−	+	u
S74B		d	u	u	u	u	u	u
S73A	SFi	d	u	u	+	u	u	u
S73B		d	+	u	u	(+)	u	u
Total+			6/9	6/6	5/6	1/9	6/6	0/0
S38A	CA3 SP	s	+	+	u	+	u	u
S38B		s	−	−	u	−	u	u
S27A		s	−	u	−	−	u	−
S31B1*^a^*	CA3d SL	s/d	+	+	+	−	u	−
S31B2*^a^*		d	+	−	+	+	u	−
S38C	CA2 SO	d	+	u	u	u	u	u
S38D		d	+	u	u	u	u	u
S38E		d	+	u	u	u	u	u
S38F		d	+	u	u	u	u	u
S38G		d	+	+	u	+	u	u
S31A1*^b^*	CA2 SR	s	+	+	+	+	u	+
S31A2*^b^*		d	−	u	+	u	u	u
S31A3*^b^*		d	+	u	+	u	u	u
Total +			10/13	4/6	5/6	4/7	0/0	1/4

Molecular profiles of presumed postsynaptic neurons, based on close apposition of axon terminals

+, Detectable positive immunoreactivity or signal; −, undetectable immunoreactivity or signal in vicinity of immunopositive signals; u, unknown (unavailable or inconclusive). Parentheses indicate weak immunoreactivity or signal. Domain, target subcellular domain (s, soma; d, dendrite).

*^a,b^*Terminals were apposed to one or all of these targets in both cases, and the targets themselves were in apparent contact; this affects the totals. As many targets were onto thin dendrites, we cannot rule out that in some cases the target dendrites originated from the same neuron. SFi, septofimbrial nucleus. PV input, PV immunopositive terminals were apposed to the target cell. Additionally tests: S74A was also CB− and SMI-32−; S74B was also SMI-32+; S27A was also CR− and CCK−; S76A-H were also ChAT− and Secretagogin−; S31B1 and S31B2 were also CCK−.

Neuron MS53_ innervated the same cortical and subcortical regions as LRN TV68a. The axon originated from the soma, traveled ventrally, and then looped dorsally, whereby several collaterals gave rise to terminals in the MS. The main axon continued via the fimbria to the septal hippocampus, where it gave rise to multiple branches, as described above. Terminals were observed at all septotemporal levels, with some arranged horizontally (e.g., in SO) and others radially (e.g., in SR). Targets in the DG were predominantly in the PML. Neuron MS103d had similar firing patterns to LRN TV68a, including “theta-off” periods (Fig. 6*a*,*b*). Terminals were observed in CA3 and DG, but the axon was weakly labeled and could not be followed to other cortical areas. We have shown that LRNs encompass several distinct kinds of septohippocampal neurons (organized by similarity in Table 2), with specialized innervation patterns and selectivity of target neurons that are complementary to the parallel HRN projections.

## Discussion

We report the innervation patterns and synaptic targets of the first medial septal LRNs in awake mice. The majority targeted the DG and CA3 and preferentially fired on the descending phase of CA1d theta oscillations when DG and CA3 principal neurons fire at the highest probability (Buzsáki et al., 2003; Mizuseki et al., 2009; Lasztóczi and Klausberger, 2017; Senzai and Buzsáki, 2017). The GABAergic LRNs innervated GABAergic interneurons. These LRNs match rat type 2 “non-rhythmic” cells coupled to the descending theta phase (King et al., 1998), taking into account electrode locations (Gaztelu and Buno, 1982; Dragoi et al., 1999). Rat type 1a and 1b rhythmically bursting neurons (Gaztelu and Buno, 1982; King et al., 1998) match the profiles of mouse Komal and Teevra cells, respectively.

Dorsal DG cells are required for spatial working memory (Niewoehner et al., 2007; Sasaki et al., 2018) and spatial activity of postsynaptic CA3 neurons is dependent on mossy fiber inputs (Sasaki et al., 2018). Neurons in the DG (Jung and McNaughton, 1993) transiently increase their cofiring during DG-dependent memory discrimination across individual theta cycles (∼100 ms) (van Dijk and Fenton, 2018) due to increased excitatory drive, disinhibition, or both. Intriguingly, for GABAergic DG-CA3-projecting LRNs that did not fire on all theta cycles, their postsynaptic targets are likely inhibited when the LRN fires or disinhibited when the LRN is silent. Indeed, the spectral content and mnemonic function of each theta cycle varies (Lopes-Dos-Santos et al., 2018). We hypothesize that the temporal dynamics of LRNs could contribute to these rapid changes through their interneuron targets. A marked reduction in LRN firing, such as during the initiation of movement, may facilitate an increase in output of their target interneurons, leading to an increase in the selectivity of the active principal neuron assemblies through increases in firing threshold. Neuromodulators released from interneurons (Katona et al., 2014), MSDB neurons (Lamour et al., 1988) or other subcortical centers (Wagatsuma et al., 2018) may contribute to recruiting specific principal cell sequences.

Medial septal LRNs strongly reduced firing during SWRs, which was typically followed by rebound spiking. Such SWR suppression was not observed for Teevra cells during similar periods of immobility (Joshi et al., 2017) and was rare for “type 2” neurons in sleeping rats (Dragoi et al., 1999). Suppression of GABAergic LRNs during SWRs would disinhibit their GABAergic targets, followed by inhibition during post-SWR rebound burst firing, thus restricting the temporal window of their participation to the SWR (Szabo et al., 2017). Some hippocampo–medial septal projecting GABAergic neurons are active during SWRs (Jinno et al., 2007; Takács et al., 2008). Reward-associated CA3 SWRs are dependent on functional mossy fiber inputs (Sasaki et al., 2018) and granule cells are recruited during SWRs (Penttonen et al., 1997). We suggest that DG-CA3-projecting LRNs are presynaptic to SWR-active interneurons, including interneurons innervating both DG and CA3 (Han et al., 1993; Szabo et al., 2017; Unal et al., 2018). We hypothesize that these presynaptic LRNs are required for the oscillatory temporal structuring of neuronal assemblies during SWRs involved in memory consolidation and reward-related behaviors.

The identity of GABAergic interneurons postsynaptic to LRNs has proved difficult to define directly, but we are able to make some predictions from the data. In CA1, GABAergic OLM and bistratified cells modulate pyramidal neuron dendrites mainly during the theta trough, when pyramidal cells as a population fire with highest probability (Mizuseki et al., 2009; Varga et al., 2012; Katona et al., 2014; Varga et al., 2014). Due to the temporal redistribution of inhibition across subcellular domains of principal neurons during each oscillatory cycle (Varga et al., 2012; Somogyi et al., 2014), the axon initial segment is disinhibited at the same time that the dendrites are maximally modulated. We hypothesize that LRNs participate in changing the firing mode of principal cells by inhibiting dendrite-targeting interneurons (Lovett-Barron et al., 2012; Szabo et al., 2017) during memory discrimination on select theta cycles containing varying spectral frequencies around the slow to mid-gamma ranges (van Dijk and Fenton, 2018; Lopes-Dos-Santos et al., 2018). In the case of PV^−^/CB^−^ LRNs, targets included CA3 PV^+^/mGluR1a^+^ neurons, which are likely to be dendrite-targeting interneurons. The multi-area innervating LRNs innervated interneuron dendrites in several cortical areas and also local MSDB neurons. The dendritic location of LRN synapses at putative sites of gap junctions between interneurons likely modulates their synchronized activity. In the case of multi-area-innervating LRNs, this effect would also synchronize or “reset” interneuron subpopulations during behavioral state changes such as the transition between quiet wakefulness and movement.

High-rhythmic Teevra cells form basket-like terminations around PV^+^/SATB1^−^ axo-axonic cells and some CCK interneurons in CA3 (Viney et al., 2013; Joshi et al., 2017). In parallel, LRNs target separate subpopulations of CA3 interneurons in addition to innervating other areas such as the DG. Further recording and labeling experiments will reveal the extent of such parallel subcortical GABAergic innervation in the cortex. It is surprising that we have not so far encountered HRN input to the DG in mouse; one rare theta-coupled PV^+^ “septo-dentate” neuron was identified in rat (Unal et al., 2018). Similar to GABAergic feedback by hippocamposeptal neurons from cornu ammonis (Tóth et al., 1993; Jinno and Kosaka, 2002; Jinno et al., 2007), there are considerable reciprocal connections between the MSDB and the DG, such as via somatostatin-expressing hilar interneurons (Jinno and Kosaka, 2002; Yuan et al., 2017).

Extensive basket-like innervation of dentate interneurons, particularly those in the ML by PV^−^/CB^−^ LRN TV78l, is likely to provide powerful inhibitory control and would be disinhibitory during SWRs. The features of PV^+^ targets on the GCL/ML border resemble axo-axonic cells (Soriano et al., 1990; Haring et al., 1997). Therefore, unlike their CA3 counterparts (Viney et al., 2013), dentate ML axo-axonic cells probably fire during SWRs. The discharge of specific granule cells during SWRs may be dependent on SWR-inhibited axo-axonic cells that target both CA3 and the DG (Han et al., 1993; Szabo et al., 2017) or through the interaction with mossy cells and hilar axo-axonic cells. The low-rhythmic firing of presynaptic GABAergic medial septal neurons to ML axo-axonic cells and the lack of high-rhythmic Teevra cell input predicts their low theta modulation. The sparse firing of granule cells during locomotion and their relatively strong theta modulation (Jung and McNaughton, 1993; Diamantaki et al., 2016; Danielson et al., 2017; Senzai and Buzsáki, 2017) suggests the participation of presynaptic rhythmically firing dendrite-targeting interneurons.

The rarity of high-rhythmic medial septal GABAergic input to the DG highlights its specialized processing compared with CA1-CA3 (Sanders et al., 2019; van Dijk and Fenton, 2018). The results so far reveal at least four kinds of cortically projecting LRNs modulating interneurons in parallel with at least two kinds of PV^+^ HRNs. The DG-projecting *CB*^+^ *LRNs* had heterogeneous firing patterns and could be differentiated based on mGluR1a immunoreactivity. The *CB*^+^*/mGluR1a*^+^ cells had higher firing rates than all other DG-innervating cells, significantly decreased their firing during SWRs, and did not show rebound firing. In contrast, the *CB*^+^*/mGluR1*^−^ neuron MS68a resembled more closely PV^−^/CB^−^ LRNs in terms of firing patterns, had the highest rhythmicity of all LRNs, and showed SWR rebound. Because we were unable to visualize the axon terminals, we could not determine whether these CB^+^ LRNs are GABAergic or glutamatergic (Wei et al., 2012; Fuchs et al., 2016). Similar to the parallel innervation of CA3 by LRNs and high-rhythmic Teevra cells, we identified an LRN innervating the RSg and PrSd, areas of the cortex that are also innervated by orchid cells. The molecular profile of this mainly RSg-projecting LRN MS104e was similar to orchid cells and the multi-area-innervating LRN TV68a. Therefore, both hippocampal and extrahippocampal areas receive functionally distinct, multiple GABAergic medial septal inputs.

How are the contributions of LRNs and HRNs coordinated? They are unlikely to interact at the level of individual cortical interneurons. Instead, in the medial septum, multi-area-innervating LRNs targeted neurons that matched the molecular profiles of other LRNs and orchid cells, but not cholinergic neurons. Orchid cells also have local axons in the MSDB with unknown synaptic targets (Viney et al., 2018). Local collaterals of Teevra cells, however, mostly innervate PV^+^/SATB1^+^ neurons (Joshi et al., 2017), which could represent multi-area-innervating LRNs or other HRNs. Both GABAergic and glutamatergic MSDB neurons are innervated by PV^+^ terminals (Henderson et al., 2010), suggesting that PV^+^ LRNs may also locally modulate the activity of glutamatergic neurons, for example, during locomotion (Fuhrmann et al., 2015). In turn, glutamatergic neurons likely depolarize both cholinergic and GABAergic MSDB neurons (Manseau et al., 2005) and also project to the cortex to provide synaptic input to both principal neurons and interneurons, including in CA3 and the DG (Colom et al., 2005; Huh et al., 2010; Fuhrmann et al., 2015). Furthermore, cholinergic neurons, which also have local axon collaterals (Wu et al., 2014), cotransmit acetylcholine and GABA, contributing to hippocampal SWR suppression (Vandecasteele et al., 2014; Saunders et al., 2015; Desikan et al., 2018; Takács et al., 2018). Therefore, coordination of rhythmic activity of LRNs and HRNs within the MSDB is probably mediated via the dynamic interactions of all three neuronal classes.

We have shown that LRNs have diverse axonal outputs, but most of them primarily target interneurons in the DG and CA3 regions, providing variable theta-modulated input and are suppressed during SWRs, allowing the activation of some hippocampal interneurons. Rhythmic activity in the brain is implemented by specialized subcortical cell types, supporting mnemonic functions by interacting locally in the MSDB and in the cortex via inhibition of select interneurons.
